# Data model, dictionaries, and desiderata for biomolecular simulation data indexing and sharing

**DOI:** 10.1186/1758-2946-6-4

**Published:** 2014-01-30

**Authors:** Julien C Thibault, Daniel R Roe, Julio C Facelli, Thomas E Cheatham

**Affiliations:** 1 Department of Biomedical Informatics, University of Utah, Salt Lake City, UT, USA; 2 Department of Medicinal Chemistry, University of Utah, Salt Lake City, UT, USA

**Keywords:** Biomolecular simulations, Molecular dynamics, Computational chemistry, Data model, Repository, XML, UML

## Abstract

**Background:**

Few environments have been developed or deployed to widely share biomolecular simulation data or to enable collaborative networks to facilitate data exploration and reuse. As the amount and complexity of data generated by these simulations is dramatically increasing and the methods are being more widely applied, the need for new tools to manage and share this data has become obvious. In this paper we present the results of a process aimed at assessing the needs of the community for data representation standards to guide the implementation of future repositories for biomolecular simulations.

**Results:**

We introduce a list of common data elements, inspired by previous work, and updated according to feedback from the community collected through a survey and personal interviews. These data elements integrate the concepts for multiple types of computational methods, including quantum chemistry and molecular dynamics. The identified core data elements were organized into a logical model to guide the design of new databases and application programming interfaces. Finally a set of dictionaries was implemented to be used via SQL queries or locally via a Java API built upon the Apache Lucene text-search engine.

**Conclusions:**

The model and its associated dictionaries provide a simple yet rich representation of the concepts related to biomolecular simulations, which should guide future developments of repositories and more complex terminologies and ontologies. The model still remains extensible through the decomposition of virtual experiments into tasks and parameter sets, and via the use of extended attributes. The benefits of a common logical model for biomolecular simulations was illustrated through various use cases, including data storage, indexing, and presentation. All the models and dictionaries introduced in this paper are available for download at http://ibiomes.chpc.utah.edu/mediawiki/index.php/Downloads.

## Background

Thanks to a dramatic increase in computational power, the field of biomolecular simulation has been able to generate more and more data. While the use of quantum mechanics (QM) is still limited to the modelling of small biomolecules [1] composed of less than a couple hundred of atoms, atomistic or coarser-grain molecular representations have allowed researchers to simulate large biomolecular systems (i.e. with hundreds of thousands of atoms) on time scales that are biologically significant (e.g. millisecond for protein folding) [2]. Classical molecular dynamics (MD) and hybrid approaches such as quantum mechanics/molecular mechanics (QM/MM) are some of the most popular methods to simulate biomolecular systems. With the explosion of data created by these simulations — generating terabytes of atomistic trajectories — it is increasingly more difficult for researchers to manage their data. Moreover results of these simulations are now becoming of interest to bench scientists to aid in the interpretation of increasingly complex experiments and to other simulators for assessing force fields and to develop coarse-grain models. Opening these large data sources to the community, or at least within collaborative networks, will facilitate the comparison of results to detect and correct issues with the methods, identify biologically relevant patterns or anomalies, and provide insight for new experiments. While the Protein Data Bank [3] is very useful as a central repository for structural data, the number of repositories for biomolecular simulations is still very limited. To the best of our knowledge the only databases that currently provide access to MD data for the community are Dynameomics [4,5] and MoDEL (Molecular Dynamics Extended Library [6]). Dynameomics and MoDEL were populated with about 11,000 and 17,000 MD trajectories of proteins respectively. One of the problems with such repositories is that the published data was generated in a specialized environment to study a given biological process (e.g. protein folding), resulting in fairly homogeneous data compared to the range of methods and software available to the community. These repositories are somewhat tied to these environments and it is uncertain how one would publish data generated outside these environments or how external systems would index or interface with these repositories. As more repositories are created the need for a common representation of the data becomes crucial to achieve semantic interoperability and enable the development of federated querying tools and scientific gateways. Note that other efforts to build repositories and scientific gateways, such as the BioSimGrid project [7] and work by Terstyanszky et al. [8], have been undertaken but so far none has been widely adopted outside their original deploying institution or organization.

In the computational quantum chemistry community, more progress has been achieved towards the development of repositories using standard data representations to enable collaborative networks. One of the main on-going efforts is led by the Quixote project [9] which aims to create a federated infrastructure for quantum chemistry calculations where data is represented with *CML CompChem* (Chemical Markup Language – Computational chemistry [10]) and integrated into the semantic web through RDF (Resource Description Framework, http://www.w3.org/RDF/). The Chemical Markup Language [11] (CML) and its computational component *CML-CompChem* aim to provide a standard representation of computational chemistry data. While the core CML XML specifies the requirements to represent molecular system topologies and properties, *CML-CompChem* supplements CML to allow the representation of computational chemistry data, including input parameters and output data (calculations). So far these extensions have mainly focused on representing quantum computational chemistry experiments as XML files. These files can be created by converting input and/or output files generated by a particular software package through file parsers such as the ones supported by the Blue Obelisk group [12] (e.g. Chemistry Development Kit, Open Babel). While *CML-CompChem* has a great potential for QM calculations [13], its usefulness for MD and biomolecular simulations in general might be limited. For example, typically trajectories of atomic positions need to be compressed or binary encoded for data movement, storage purposes, and/or accuracy. Embedding this information into a verbose XML file such as CML will not be the optimal solution, at least not for the description and formatting of the raw output. Another obstacle to the conversion of MD experiments to a single-file representation is the common definition of many separate input files (e.g. system topology, method parameters, force field) necessary to prepare an MD simulation and define the different iteration cycles (e.g. minimization, equilibration, production MD). In quantum chemistry, the targeted molecules and calculation parameters are typically defined in a single input file (e.g. “.com” file for Gaussian [14] and “.nw” file for NWChem [15]) which makes this conversion much simpler. The output files generated by quantum chemistry software packages usually already contain the final results the user is interested in while in MD the raw output – i.e. multiple files containing the trajectories of atomic positions, energies and other output information – has to be further processed through various analysis tasks to create meaningful information. These post-processing steps involve the creation of new input and output files, making the conversion of an experiment to a single XML file even more difficult.

Perhaps one of the main barriers to build repositories for biomolecular simulations is the lack of standard models to represent these simulations. To the authors’ knowledge no published study has assessed the needs of the community regarding biomolecular simulation repository data models. Therefore it is unclear which pieces of information are considered essential by researchers and how they should be organized in a computable manner, so that users can:

– Index their data and build structured queries to find simulations or calculations of interest, not only via the annotations, but also with access to the raw data (files).

– Summarize, present, and visualize simulation data either through a web portal or more static documents (e.g. PDF document, XML file).

These models should be designed to include not only the description of the various independent computational tasks performed but also a high-level description of the overall simulated experiment. Each experiment can be related to multiple concepts that help understanding what was simulated, how, and in which context. These concepts can be grouped into the following categories:

– *Authorship*: information about the author, grants and publications related to the experiment

– *Methods*: computational method description (e.g. model building, equilibration procedure, production runs, enhanced sampling methodology) and associated inputs / parameters

– *Molecular system*: description of the simulated molecules from a structural, chemical, and biological point of view

– *Computational platform*: description of the software used to run the computational tasks, the host machine (computational environment), and execution configuration

– *Analysis*: derived data that can be used for quality assessment of the simulations

– *Files*: information about the raw simulation input and output files, such as format, size, location, and hosting file system

In this study we describe our efforts to formalize the needs of the community regarding the elements necessary to index simulation data. This work was initiated in part to support the iBIOMES (Integrated BIOMolEcular Simulations) project [16], an effort to create a searchable repository for biomolecular simulations, where the raw data (input and output files) is made available so that researchers can rerun the simulations or calculations, or reuse the output to perform their own analysis. In the initial prototype a set of software-specific file parsers were developed to automatically extract common data elements (metadata) and publish the raw data (i.e. the input and output files) to a distributed file system using iRODS (integrated Rule-Oriented Data System [17]). The published files and collection of files (experiments) are indexed based on the extracted data elements and are stored as attribute-value-unit triplets in a relational database. In this paper we introduce a list of common data elements and a data model that will help iBIOMES and future biomolecular simulation data repository developments move towards semantic interoperability.

### Motivation for a common data representation: examples

The development of a common framework for data representation provides users with a large amount of flexibility to develop new tools for managing the data while maintaining interoperability with external resources. In this section we present three different examples that demonstrate the need for a standard representation of biomolecular simulation data, whether it is for indexing or presentation to the user. All three examples have been implemented to some extent in prototype form here. The first example is based on our experience with iBIOMES [16], where simulation-specific metadata is associated at the file or directory level, through a specialized file system (iRODS [17]). The second example shows how one would use a model-based approach to build a repository where simulation parameters and provenance metadata are stored in a relational database. Finally the last example illustrates how a model-based API (Application Programming Interface) can be used to automatically generate XML and HTML summaries for the simulations being published.

#### Example 1: building a repository based on file annotations

One of the simplest ways to index simulations is to tag the associated files and directories with user annotations summarizing their content. These tags can be simply stored in a database or indexed via dedicated systems such as Hadoop [18,19] or Apache Lucene [20]. This approach is well suited for fast searches based on keywords or attribute-value pairs. In the iBIOMES system [16] these tags are managed by the iRODS framework [17], which enables the assignment of attribute-value-unit triplets to each file and directory in a distributed file system. This approach is very flexible since it allows the use of tags that represent common concepts such as computational methods and biological features, and user- or lab-specific attributes as well. In iBIOMES, a catalogue of common attributes was defined for users to annotate their data. The definition of such attributes is important as they can be tied to actionable processes, such as analyses, visualizations, and ultimately more complex workflows. It is then possible to build a user interface that presents the data and performs certain actions based on the existence of certain attributes or their associated values. For example if the format of a file is PDB (File format = “PDB”), then the user interface could enable 3D rendering of the associated molecules through Jmol [21]. A data dictionary that would offer possible values for a particular attribute is important as well. Each term should be well defined to leave no ambiguity to the user. A dictionary of force fields for example could list all the common force fields with a textual description, a type (e.g. classical, polarizable, coarse-grained), and the associated citations for each entry. A catalogue of common data elements, associated to a data dictionary, is also useful for users to pick from to facilitate annotations and build queries. The catalogue used in iBIOMES was defined internally by our lab and probably is not yet sufficiently exhaustive for the community at large. However, creating a catalogue of common data elements (CDE) supported by the community is a first step towards the standardization of biomolecular simulation data description. Defining a subset as recommended (i.e. the core data elements) would go a step further and set a criterion to assess the quality of the data publication process. Finally, linking these CDEs to existing terminologies or ontologies would bring semantic meaning to the annotations, enabling data discovery and query via external systems.

#### Example 2: building a repository based on a relational database

**Figure 1:**
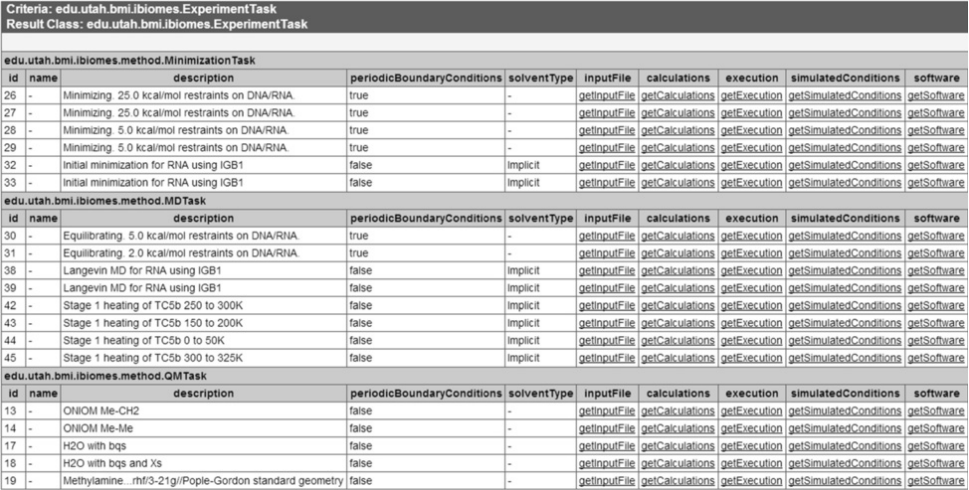
**Screenshot of the web interface generated via the caGrid tools.** The screenshot presents a listing of the computational tasks that were published into the caGrid test system. The user request was automatically translated into a SQL query via Hibernate to return the rows form the tables mapping to the class *ExperimentTask* and its child classes *MinimizationTask* (minimizations), *MDTask* (MD runs), and *QMTask* (QM calculations). For each row, a set of *get* methods (e.g. getSoftware) link to the associated objects for more details (e.g. Software name and version).

While a CDE catalogue is important, it lacks the representation of relationships between elements unless it is linked to a well-structured taxonomy. For example, if a user is interested in simulations of nucleic acids, a hierarchical representation of biomolecules could be used to infer that the user is actually looking for any simulation of DNA or RNA. The aim of a logical data model is to give a representation of the domain that captures the business needs and constraints while being independent from any implementation concern [22]. Such a model can provide the foundations for the design of a database and can be used to automatically generate API skeletons using modern modelling tools (e.g. Enterprise Architect, ArgoUML, Visual Paradigm). Since it is a domain-specific representation of the data, it can also serve as a starting point to develop a terminology or ontology specific to this domain. In this second example we demonstrate how a data model could be used to prototype a repository for biomolecular simulations where simulation parameters and provenance metadata are organized and stored in a relational database. We created a UML (Unified Modeling Language, http://www.uml.org/) model including logical and physical entities to build a relational database that could eventually be wrapped as a Grid service. The Grid [23] represents a great infrastructure for collaboration because of the underlying authentication scheme and data discovery services available, but also because of the semantic and syntactic integration. For this example we decided to mock up a data grid service using the caGrid [24] framework. caGrid was supported by the National Cancer Institute (NCI) and aimed to create a collaborative network for researchers to share cancer data, including experimental and computational data. The caCORE (cancer Common Ontologic Representation Environment) tools that were developed in this context facilitate the creation of the grid interfaces by automatically generating the necessary Java code from a UML model. These tools are now maintained by the National Cancer Informatics Program (NCIP) and available at: https://github.com/NCIP/. For this example we mapped the logical model to a data model using the *caAdapter* graphical tool. The final UML model and database creation scripts for MySQL (http://www.mysql.com/) are available for download at: http://ibiomes.chpc.utah.edu/mediawiki/index.php/Downloads. More details about the UML model are provided in the section introducing the logical data model. The caCORE SDK (Software Development Kit) was then used to generate the *Hibernate* (http://www.hibernate.org/) interfaces to the database along with a web interface that can be used to create simple queries or browse the published data. A screenshot of the generated interface is given in Figure 1 (listing of various published computational tasks). To actually build and deploy the data service onto a Grid, one would have to use the *Introduce* module. Semantic integration is also possible via the *Semantic Integration Workbench* (SIW), which enables tagging of the domain model with concepts from standard terminologies (e.g. ChEBI, Gene Ontology).

#### Example 3: representing experiments using XML

**Figure 2:**
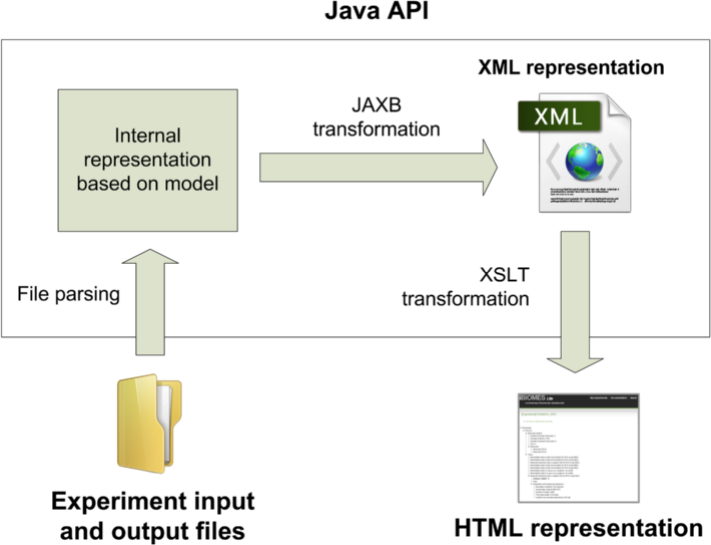
**Generating an XML representation of experiments using a Java API.** The Java API is used to parse the input files and create an internal representation of the virtual experiment as a set of computational tasks. JAXB is then used to generate an XML representation of this internal model, while XSLT is used to perform a last transformation into a user-friendly HTML page.

While a database provides a single endpoint to query data, other types of data descriptors become necessary when moving data between file systems, or simply to provide a light-weight description of the data. XML has been widely adopted by the scientific community to represent structured data because of its flexibility and support by web technologies. In the field of computational chemistry *CML-CompChem*[10] aims to provide a detailed representation of computations but currently lacks support in the molecular dynamics community. *BioSimML* (Biomolecular Simulation Markup Language [25]) was developed specifically for biomolecular modelling and supports QM/MM simulation representations but its current status in uncertain. The Unified Molecular Modeling (UMM) XML schema [26] is currently being developed by ScalaLife (Scalable Software for Life Sciences, http://www.scalalife.eu/) and will attempt to provide a detailed description of MD runs, so that these files can be used as a standard input to run within various MD engines. So far these XML-based formats have focused on giving a low-level representation of the simulation runs so that data can be converted between legacy formats. In this example we generate an XML-based representation of the experiment as a whole (multiple tasks), with a limited granularity for the description of each task. For this purpose we developed a Java API based on our logical model to generate XML representations of experiments (Figure 2). Format-specific file parsers developed for the iBIOMES project [16] read in input and output files associated to an experiment to create an internal representation of the experiment and associated computational tasks. In the Java code, classes are annotated with *Java Architecture for XML Binding* (JAXB, https://jaxb.java.net/) annotations to map the logical model to an XML schema. The JAXB API can then be used to automatically output XML documents based on the internal Java representation of the experiment or read in an XML file to build the Java objects. The same process could be implemented in various languages, using *CodeSynthesis XSD* (http://www.codesynthesis.com/products/xsd/) in C++ or *PyXB* (http://pyxb.sourceforge.net/) in Python for example.

**Figure 3:**
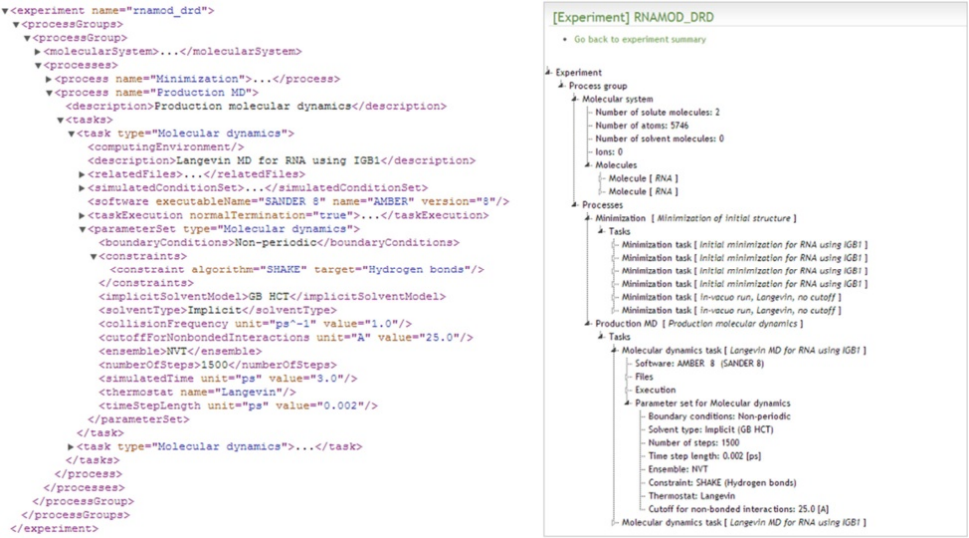
**XML and HTML-based representations of an experiment.** Auto-generated XML sample (left) and corresponding HTML tree view (right) representing the different tasks run for an MD study of RNA using the AMBER software package.

The XML output does not aim to be sufficient to recreate input or output files in legacy formats but it will provide enough information for users to rapidly understand the computational methods and structures represented by the associated raw data. This type of XML document can be used as a way to give a detailed summary of experiments when exchanging data, compressed with the raw data for example. These documents can be transformed through XSLT (eXtensible Stylesheet Language Transformations) to be rendered as HTML pages and build repository web interfaces. A sample XML output along with an HTML-based tree view generated through XSLT are presented in Figure 3. For this example a set of AMBER-specific [27] file parsers was used to parse a directory containing all the input and output files associated to an MD study of RNA. Common data elements related to the molecular system topology were extracted from the AMBER parameter/topology file while task (minimization and MD runs), parameter set (e.g. implicit solvent, number of iterations), and computational platform information were extracted from the AMBER MD output files.

#### Summary

These three prototypes serve as examples demonstrating the need for a catalogue of CDEs and the representation of relationships between concepts through a data model. The catalogue of CDEs, associated to a data dictionary, provides the basis for a controlled vocabulary that can be used to annotate experiment data (e.g. files and directories) and build queries. The data model provides extra information as it links concepts together and allows more complex and structured queries, through a relational database for example. The second example showed how modern software engineering tools can use data models to generate database schemas and APIs for repository developments. Finally the last example showed that XML representations can be easily generated if the API follows a model-based approach.

In this paper we introduce a list of CDEs built upon community feedback, and a logical model that ties dictionaries and common data elements together. Common data elements for simulation data indexing and presentation were identified through a survey, while recommendations are made for trajectory and analysis data description. The common data elements were organized through a logical data model, which was refined to include dictionaries and minimize data redundancy. Finally the design and implementation for a subset of these dictionaries is introduced.

## Experimental

### Identification of core data elements

#### Survey

A survey was distributed to the community to assess the list of data elements that was defined in iBIOMES [16]. This initial list of common data elements was based on the BioSimGrid [7] data model and supplemented with new elements to reflect the needs of our lab and various collaborators at the University of Utah, and to add descriptions of quantum chemistry calculations. The main goal of the survey was to identify which elements were missing and which ones were not so important according to the community. A list of 47 data elements describing simulation runs and the associated files was presented to experts. These data elements were grouped into 6 categories for organizational purpose: authorship (user information and referenced citations related to a particular run), platform (hardware/software), molecular system (molecules being studied, independently from the model chosen), molecules (info about the molecules composing the system), methods (can apply to any method, including QM and MD), molecular dynamics, and quantum mechanics. The experts were asked to score the data elements based on how important they are to them to describe their own data and/or to index community data and build search queries. Scoring was based on a Likert scale (1 = “Not important at all”, 2 = “Not very important”, 3 = “Not sure, 4 = “Important”, 5 = “Very important”, and “N/A” for non-applicable). In each group, the experts were also allowed to propose missing data elements and/or comment on the listed elements.

The survey was made available online (see extract in Additional file 1) in March 2012 for about a month and promoted through the Computational Chemistry List (CCL) and the AMBER developers’ mailing list. The CCL list is a fairly well known group for general discussions related to computational chemistry, perhaps with an emphasis on QM-related methods. The AMBER developers group represents a variety of theoretical disciplines (MD, QM, QM/MM), with developments targeting various types of systems (e.g. proteins, nucleic acids, lipids, carbohydrates, small compounds) and discussions on how to best use the software, methods and force fields. Individual emails were also sent to different research groups at the University of Utah that are specialized in computational chemistry.

#### Trajectory and analysis data

The survey did not include any analysis- or file-related data elements. The Dublin Core metadata (http://dublincore.org/documents/dces/) can be used as a good reference to describe files at a high level (e.g. author, format). Analysis data on the other hand is very complex to describe because of its direct relation to the raw data it derives from (e.g. use of multiple input files representing experimental and computed data) and the existence of numerous analysis methods that can be problem-specific (e.g. Protein vs. RNA, QM vs. MD). In most cases it will not make sense to use analysis data to index an experiment either. For example looking for MD trajectories with a particular RMSD (root mean square deviation) value would be irrelevant without providing more context about the system and the method used to calculate the value. Although analysis data is a key factor to assess the quality of a simulation, its use for data indexing and retrieval is not trivial and therefore was not included in the survey. A generic framework for the description of trajectory and derived data is nevertheless provided in the *Results* section.

### Logical model

#### Overview

The logical model presented here was derived from a conceptual model that organized all the identified common data elements into a defined domain. The conceptual model was reduced into a logical model with the assumption that the raw input and output files are made available (in a repository similar to iBIOMES or MoDEL) and that the model would be used to index the data rather than providing a complete view of the results (e.g. calculation output, structures defined in each MD trajectory frame). Although analysis data and quality criteria are crucial to provide an objective perspective on experiment results, no associated concept was included in the current model. The granularity of the model was limited to a sufficient level of details that makes it computable. For example, the description of the theory behind modelling methods is not part of the model. The end-goal being to share the results of the simulations or calculations with the community we limited our model to include only popular methods that are used for the study of biomolecules or smaller ligands.

#### Use of dictionaries

One of the main features of this logical model is the integration of dictionaries to avoid data redundancy. For example a dictionary containing definitions of force fields (e.g. name, type, citations) can be referenced by molecular dynamics tasks, instead of creating individual force field definition entries every time the force field is used. The integration of dictionaries into the model should not enforce mappings to standard definitions but rather enable links between specific values and standard definitions only if they exist. If no mapping exists the user should still be able to publish the data. This is achieved through the storage of “specific names” outside the dictionaries with an optional reference to the term definition, where the standard version of the name (not necessarily different) is defined. For example if the basis set “LANL2DZ” is used in a QM calculation, but no corresponding entry exists in the basis set dictionary, the name of the basis set will still be stored in the database when publishing the data to allow queries on the calculation.

#### Units

Certain attributes need to be associated to a unit to be understood by a human or a computer. Different software packages might use different units to represent the same attribute. For example, distances in AMBER [27] are measured in Ångströms while GROMACS [28] uses nanometres. When publishing data to a repository one should either convert the values using units previously agreed upon or make sure that the units are published along with the values. In both cases, mechanisms should be in place to provide a description of the units when pulling data from the repository. For the description of this model we assume that the units are already set in the repository, therefore they are not included in the description of the model.

### Dictionaries

While most of the data described in a logical model for biomolecular simulations can be directly parsed from the input and output files, dictionaries containing standard definitions and values for certain data elements need to be prepopulated. In this paper we present the design and implementation of several dictionaries that can be used to facilitate data publication and queries. For example, if a user is interested in QM calculations based on Configuration Interaction (CI) theory, a dictionary of all CI methods will be needed to return all the calculations of interest (e.g. CISD, CISD(T)). Another interesting use of these dictionaries is within the code of the file parsers. Instead of defining standard values within the code, one can use these dictionaries to lookup information on the fly, and possibly use it to publish the data into the target repository.

An initial set of dictionaries was populated using the BiosimGrid [7] database dictionaries (source code available at: http://sourceforge.net/projects/biosimgrid/). They were then refined internally and supplemented with new dictionaries, especially to include QM-related definitions (e.g. basis sets, QM Methods).

## Results

### Identification of core data elements

#### Survey

At the closing of the survey we were able to collect 39 responses (20 through CCL, 10 through the AMBER developers list, and 9 through emails). The results of the survey are presented in Additional file 2. The respondents listed a few data elements they felt were missing from the proposed list or that needed to be refined (see comments in Additional file 3). For instance, in the authorship category, a data element representing research grants was missing. For the representation of the molecular system, data elements representing important functional groups of the solute molecules should be added, along with, optionally, the apparent pH of the solvent. Adjustments should also be made to distinguish the different species in the system, and flag them as part of the solvent or the solute. For the computing environment information, a respondent showed interest in knowing whether the software package is compiled in single, double, or mixed precision, what the memory requirements are for a run, and even what parallelization scheme is used. All these elements are very technical and might interest only a very limited number of users, even in the developer’s community. The notion of hardware architecture was not clearly defined in the survey since it should have already included the use of GPU (see comment in Additional file 3). A better representation of the hardware architecture can be done through three different data elements: the CPU architecture (e.g. x86, PowerPC), the GPU or accelerator architecture (e.g. Nvidia GeForce GTX 780, AMD Radeon HD 7970, Intel PHI), and possibly a machine or supercomputer architecture identification (e.g. Cray XK7, IBM Blue Gene/Q, commodity Infiniband cluster, etc.) and name (stampede.tacc.utexas.edu, h2ologin.ncsa.illinois.edu, keeneland.gatech.xsede.org, etc.). For the computational methods, data elements were missing for the representation of both MD and QM-specific parameters. In QM, the following elements were missing: exchange-correlation functionals (for DFT), pseudopotentials and plane wave cut-offs, and whether frozen core calculations are performed or not. Some comments pointed the fact that the notion of convergence can be very subjective, especially when dealing with MD trajectories where multiple minima (conformations) can be found over time (see comments in Additional file 3). The convergence flag and criteria were assigned as QM-specific data elements to reflect this. For MD, the context of the run (i.e. whether it is a minimization, an equilibration, or a production run) was missing. Representations of restraints and advanced sampling methods (e.g. replica-exchange, umbrella sampling) were also missing. More detailed properties were listed by the respondents. These included the order of expansion for LINCS-based constraints and the order of interpolation for Particle-Mesh Ewald. At this point it is not clear if such parameters need to be tracked since users would hardly use these to create queries and we assume that they can be directly read from the raw input files if necessary.

Based on the results of the survey and the various comments of the community we propose a set of common data elements for biomolecular simulation data indexing, listed in Additional file 4. The table reorganizes the granularity of the identified elements by making a distinction between data elements (concepts) and attributes (properties). For example the barostat data element has at least one property: an implementation name (e.g. “Andersen”, “Berendsen”). Depending on the type of barostat other properties could include a time constant and a chain length (e.g. Nose-Hoover barostat). We also included “derived” properties that would be inferred from other properties if the right terminology or dictionary is available. For example, the name of a QM method (e.g. MP2, B3LYP) should be enough to infer the level of theory (e.g. Møller-Plesset, DFT), and the name of the force field (e.g. AMBER FF99SB) should be sufficient to infer its type (e.g. classical). This distinction is important as it can help the developers choose which properties should be actually stored (e.g. in a database or an XML file) and which ones could be inferred. The set also contains recommended and optional data elements/attributes. An attribute is marked as recommended if its average score (i.e. the sum of Likert scale scores divided by the number of responses for that element) is greater than 4.0 (“Important”), otherwise it is marked as optional. Attributes proposed by the respondents were categorized through an internal review performed by our lab, composed of researchers running molecular dynamics simulations and quantum chemistry calculations on a daily basis. A data element is considered recommended if it has at least one recommended attribute. The current list contains 32 data elements and 72 attributes (including 30 recommended attributes).

We recognize that the process by which the data elements were defined and characterized is not perfect. Although the number of respondents was fair (between 37 and 39 depending on the data element), certain data elements had to be added or redefined based on an internal review by some of our lab members, which might have created some bias towards the needs of our lab rather than a general consensus in the community. Despite these limitations the list of data elements proposed here may be considered the first attempt to summarize the needs of the computational chemistry community to enable biomolecular simulation data indexing and queries. This list should be a good starting point to create a list of standard metadata to tag files using simple attribute-value pairs or attribute-value-unit triplets, as it is the case for iBIOMES via the iRODS metadata catalogue [17]. Although this list is fairly exhaustive, it is not complete and we hope that by publishing it the community will be able to provide more feedback and build on it, with the intent of this data model being extensible. The list is available on the iBIOMES Wiki at: http://ibiomes.chpc.utah.edu/mediawiki/index.php/Data_elements. Field experts who want to contribute to the list can request an account on the wiki.

#### Trajectory files

In most MD software packages the computed trajectories of atomic coordinates are stored in large files (~MB-TB) with each containing one or multiple time frames (e.g. PDB, AMBER NetCDF, DCD). This is the raw data that repositories would actually store and index for retrieval. Until now we have been focusing on the description of the computational tasks that were used to generate this data, i.e. the provenance metadata. This metadata can be used to find a given experiment and all associated trajectory files. On the other hand new attributes need to be assigned at the trajectory file level to describe their content and ultimately enable automatic data extraction and processing by external tools (e.g. VMD [29], CPPTRAJ [30], MDAnalysis [31]). Such attributes include the number of time frames, time between frames, number of atoms in the system and/or reference to the associated topology file, presence or absence of box coordinates, velocity information, and so on. It is important to note that the use of self-descriptive formats such as NetCDF (http://www.unidata.ucar.edu/software/netcdf/) would allow trajectory files to carry not only the description of the dataset, but also the provenance metadata, for example using the CDEs previously defined. Perhaps one of the most important attributes to give context within a full experiment is the index of a trajectory file within the set of all trajectory files representing a given task or series of tasks. Although self-descriptive formats could easily keep track of this information, it is non-trivial to generate such an index as tasks can be run independently outside of a managed workflow such as MDWeb [32], which would be able to assign these indexes at file creation time. The order of trajectory files is therefore commonly inferred from their names (e.g. “1.traj, 2.traj, 3.traj”). This approach usually works well although some errors might occur when trying to automate this ordering process. For example “10.traj” would be ranked before “2.traj” if a straight string comparison is performed (vs. “02.traj”). Strict naming conventions for trajectory data (raw, averaged, and filtered on space or time) should help circumvent these problems.

#### Analysis data

Although some analysis tasks are common to most biomolecular systems for a particular method (e.g. RMSD calculations of each frame in the trajectory to a reference structure) the number of analysis calculations one can perform is virtually infinite. There is currently no standard to describe the output of the analysis. Some formats might enable the description of the values (e.g. simple CSV or tab-delimited file with labelled columns and/or rows) but more structured files are required to describe the actual analysis process that generated the set of values contained in the file. Formats such as NetCDF are adapted to store this kind of description but are not commonly used to store biomolecular simulation analysis data. Instead comma- or tab-delimited files formats are usually preferred for their simplicity, readability, and support by popular plotting tools (e.g. MS Excel, OpenOffice, XmGrace). Assuming that the dataset is physically stored in such a file or in a relational database, a minimal set of attributes should be defined to facilitate reproduction of the analysis, as well as enable reading and loading into visualization tools with minimal user input. We believe that the strategy used in the NetCDF framework to break down data into variables with associated dimensions is a simple and logical one, and so we follow a similar strategy here.

– *Data dimensions*: Defines dimension sizes for defined data sets (i.e. variables). Any number of dimensions (including zero if data is scalar) can be defined.

– *Data variables*: The actual data. Report type (e.g. integer, float), labels, and units for all the values contained in a given set. One or more dimensions can be associated with a given variable based on its overall dimensionality. Zero dimensions correspond to a single value (e.g. average RMSD value), one dimension is an array (e.g. RMSD time series), two dimensions are a matrix (e.g. coordinate covariance), etc.

Another set of attributes need to be defined to represent the provenance metadata, i.e. how the analysis data was derived from the raw trajectories. Although different analysis tasks will require different input data types and parameters, a list of common attributes can be defined to provide a high-level description of the analysis task:

– Name (e.g. “RMSD”) and description (“Root mean square deviation calculation”) of analysis method (see entries defined in our MD analysis method dictionary)

– Path to the input file describing the task (if applicable).

– Name and version of the program used, along with the actual command executed.

– Execution timestamp

– Reference system, if any (self, experimental, or other simulated structure)

While these attributes might not be sufficient to automatically replicate the results they should provide enough information for users other than the publisher to understand how the analysis data was generated and how the analysis task can be replicated.

A further set of attributes can be defined to provide additional details on the scope of the analysis and describe in detail the data from which the current data has been derived:

– File dependencies

– Filter on time

– Filter on space (e.g. heavy atoms only, specific residue)

These would facilitate maximum reproducibility as well as enable detailed searches on very specific types of analysis. The ‘File dependencies’ attribute may include information like the trajectory used in a given calculation, which could also be used to check if the current analysis is up-to-date (e.g. if the trajectory file is newer than the analysis data, the analysis can be flagged as needing to be updated). The ‘Filter on time’ attribute might describe a specific time window or subset of frames used in the analysis. Since these attributes are perhaps not as straightforward for analysis programs to report as the other attributes, they could be considered optional and/or set by the user after the data is published. The ‘Filter on space’ attribute could be particularly useful, since it would allow one for example to search for all analyses of a particular system done using only protein backbone atoms or only heavy atoms, etc. However, this would require translation of each individual analysis program’s atom selection syntax to some common representation, which is no small task and would increase the size of the metadata dramatically for certain atom selections. In many cases it is likely that the atoms used in the analysis could be inferred from the command used, so this attribute could also be considered optional. Two examples of how these attributes might be applied to common analysis data are given in Additional file 5.

### Logical model

#### Overview

**Figure 4:**
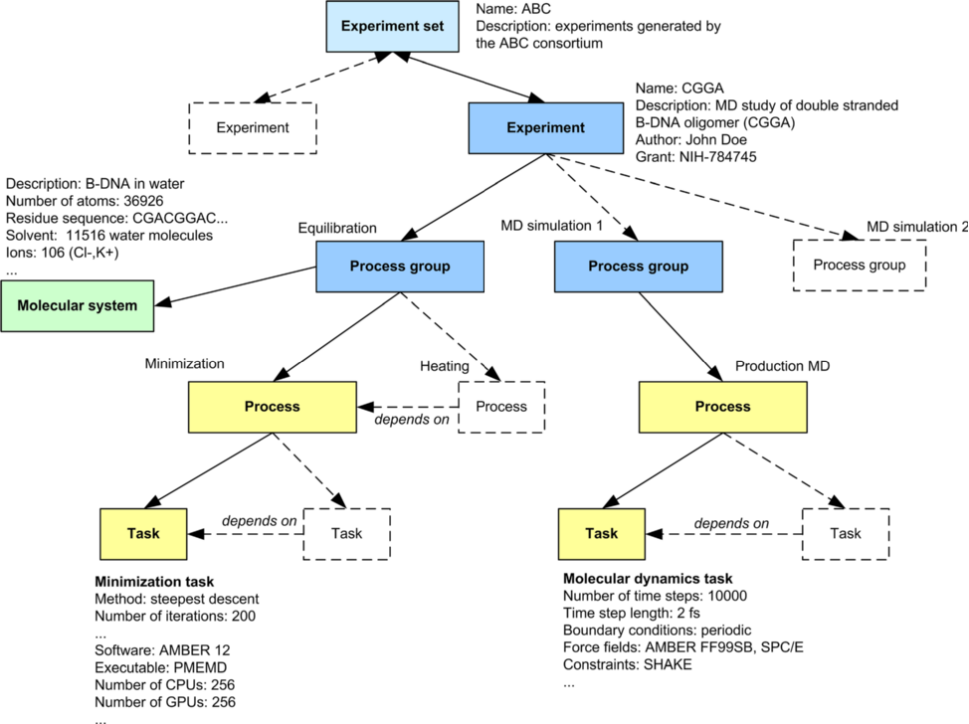
**Illustration of the data model used to represent virtual experiments.** Each experiment is a set of tasks, grouped into processes (e.g. minimization, equilibration, production MD) and process groups applied to the same molecular system (e.g. B-DNA oligomer).

In this model the central concept is the *virtual experiment*, a set of dependent computational tasks represented by several input and output files. The goal of this model is to help create a common description of these *virtual experiments* (stored in a database or distributed file system for example) for indexing and retrieval. The overall organization of *virtual experiments* is illustrated in Figure 4. For the rest of this paper *virtual experiments* will be simply denoted as *experiments*. The organization of an experiment as a list of processes and tasks was inspired by the *CML-CompChem*[10] schema. In *CML-CompChem* the job concept represents a computer simulation task and can be included into a series of consecutive sub-tasks designated as a job list. The concepts of *experiment*, *process group*, *process*, and *task* are introduced here to handle the representation of tasks that might be run in parallel or sequentially, and that might target the same or different systems. An experiment *process group* is defined as a set of computational processes targeting the same *molecular system*, where a *process* is defined as a set of similar tasks (e.g. minimization tasks, MD tasks, QM tasks). In MD, the minimization-heating-production steps can be considered as a single *process group* with 3 different *process* instances. If multiple copies of the system are simulated, each copy will be considered a separate *process group*. In QM, a *process* would represent a set of sequential calculations on a compound. If various parts of the overall system are studied separately (e.g. ligand vs. receptor), each subsystem should be assigned to a different *process group*.

Within the scope of an experiment, multiple tasks and group of tasks will be created sequentially or in parallel, and based on intermediate results. To keep track of this workflow, dependence relationships (*dependencies*) can be created between tasks, between processes, and between process groups.

#### Notations

In the following sections we present the overall organization of the model through an object-oriented approach where the concepts (e.g. experiments, tasks, parameter sets, and molecular systems) are represented by classes with attributes. The description is supported by several class diagrams using the UML notation. For example inheritance is characterized through a solid arrow with an unfilled head going from the child to the parent class. Along with standard UML notations, we defined the following colour scheme to guide the reader:

– Blue: classes giving a high-level description of the experiments and tasks

– Yellow/orange: method/parameter description

– Green: classes describing the molecular system independently from the computational methods

– Pink: classes related to authorship and publication (e.g. citations, grants)

– Grey: description of the hardware or software used to run the tasks

Finally, classes representing candidates for dictionary entries are marked with wider borders.

#### Experiments, processes, and tasks

**Figure 5:**
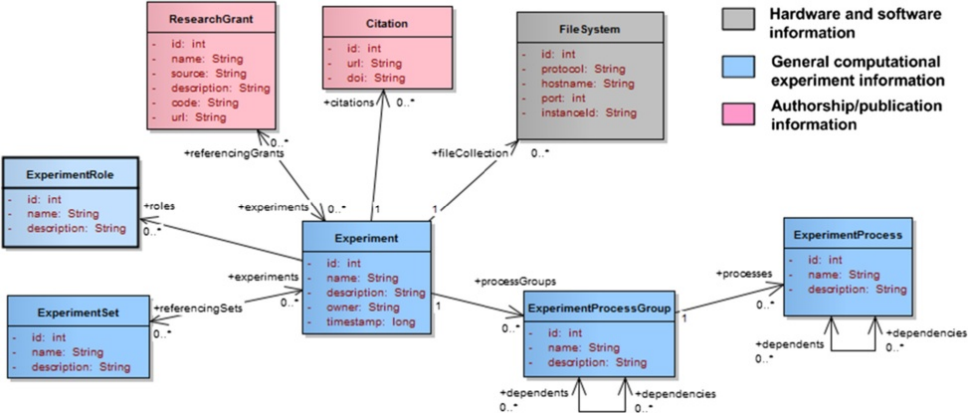
Concepts used to describe the context of the experiments.

Figure 5 presents the concepts that can be used to describe the context of an *experiment*. Each *experiment* can be given a *role*, i.e. the general rationale behind the experiment. Examples of *experiment roles* include simulation (dynamics), geometry optimization, and docking. These roles should not be associated to any computational method in particular. Each *experiment* can be linked to a particular *author* (including institution, and contact information) to allow collaborations between researchers with common interests. Publications related to a particular experiment (*citations*) or that use the results of the experiments can be referenced. *Grant* information is important as well since it allows researchers to keep track of what their funding actually supports.

*Experiment sets* (Figure 2) are collections of independent *experiments* that are logically associated together, because of similar context (e.g. study of the same system using different methods) or simply for presentation purpose or to ease retrieval by users (e.g. all the experiments created by a certain working group). An *experiment* can be assigned to multiple *experiment sets*.

**Figure 6:**
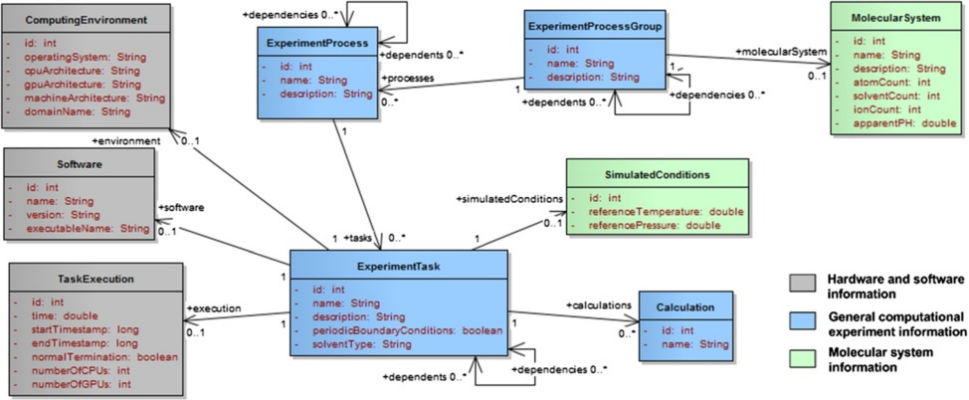
Description of experiments, processes, and tasks.

An *experiment task* corresponds to a unique computational task defined in an input file. Figure 6 presents the main concepts associated to *experiment tasks*. These include the definition of the actual *calculation* (e.g. frequency calculation and/or geometry optimization in QM, whether the dynamics of the system are simulated), the description of the *simulated conditions* (reference pressure and temperature), and the definition of the method (e.g. QM, MD, minimization) and input parameters (e.g. basis set, force field). More details about the different types of *tasks* and simulation parameters are given in the computational method section. Each *task* is executed within a *computing environment, i.e.* the set of hardware and software components used to run the simulation software package. These components include the operating system, the processor architecture, and the machine/domain name. Information about the *task execution* within the *computing environment*, including *execution time, start* and *end timestamps,* and *termination status* can be tracked as well. The *software* information includes *name* (e.g. “AMBER”) and *version* (“12”). In certain cases a more specific name for the *executable* is available. This can provide extra information about the compilation step and/or the features available. In Gaussian [14], for example, this information can be found in the output files: “Gaussian 09” would give a generic version of the software package while “EM64L-G09RevC.01” would give the actual revision number (“C.01”) and the target architecture of the executable (e.g. Intel EM64). For AMBER, the executable name would be either “SANDER” (Simulated Annealing with NMR-Derived Energy Restraints) or “PMEMD” (Particle-Mesh Ewald Molecular Dynamics), which are two alternatives to run MD tasks within the software package.

### Computational methods

**Figure 7:**
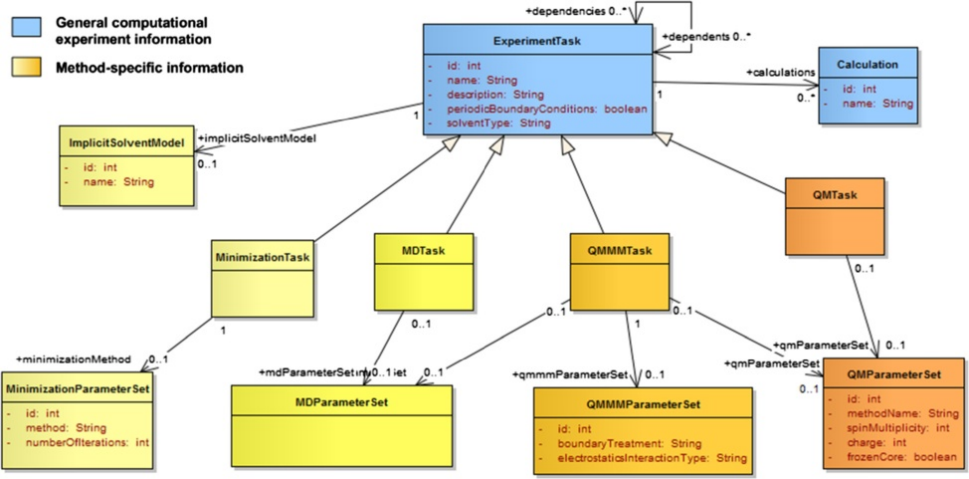
Organization of computational methods into tasks and parameter sets.

The most common methods for biomolecules include QM, MD, and hybrid QM/MM. In this model we focus on these methods but we allow the addition of other methods by associating each *task* to one or multiple *parameter sets* that can be combined to create new hybrid approaches. This decomposition was applied to MD, minimizations (e.g. steepest descent, conjugate gradient), QM, and QM/MM methods as illustrated in Figure 7.

### Method-specific tasks and parameter sets

Common attributes of any computational method are represented at the *ExperimentTask* level. These include names (e.g. “Molecular dynamics”), description (e.g. “new unknown method”), types of boundary conditions (periodic or not), and the type of solvent (in vacuo, implicit, or explicit). Method-specific *tasks* (*MinimizationTask*, *MDTask*, *QMTask*, *QMMMTask*) are created to capture the parameters that would not be shared between all methods. Simulation parameters include any parameter related to the method or task that would be set before a simulation is run. These parameters are aggregated into sets that can be reused between methods. For example, the MD-specific task (*MDTask*) references *MDParameterSet*, which includes the definitions of the *barostat*, *thermostat* and *force fields*. The QM/MM-specific task (*QMMMTask*) references the same *parameter set* since these definitions are necessary to describe the computational method to treat the MM region. It also references a *QM-specific parameter set* to describe the QM method and a *QM/MM-specific parameter set* to describe the treatment of the QM/MM boundary. A new task type could be created for multi-level quantum calculations. In this case the task would reference multiple *QM parameter sets* and a new type of parameter sets that would define at least the algorithm or implementation used to integrate the different levels (e.g. ONIOM [33]).

**Figure 8:**
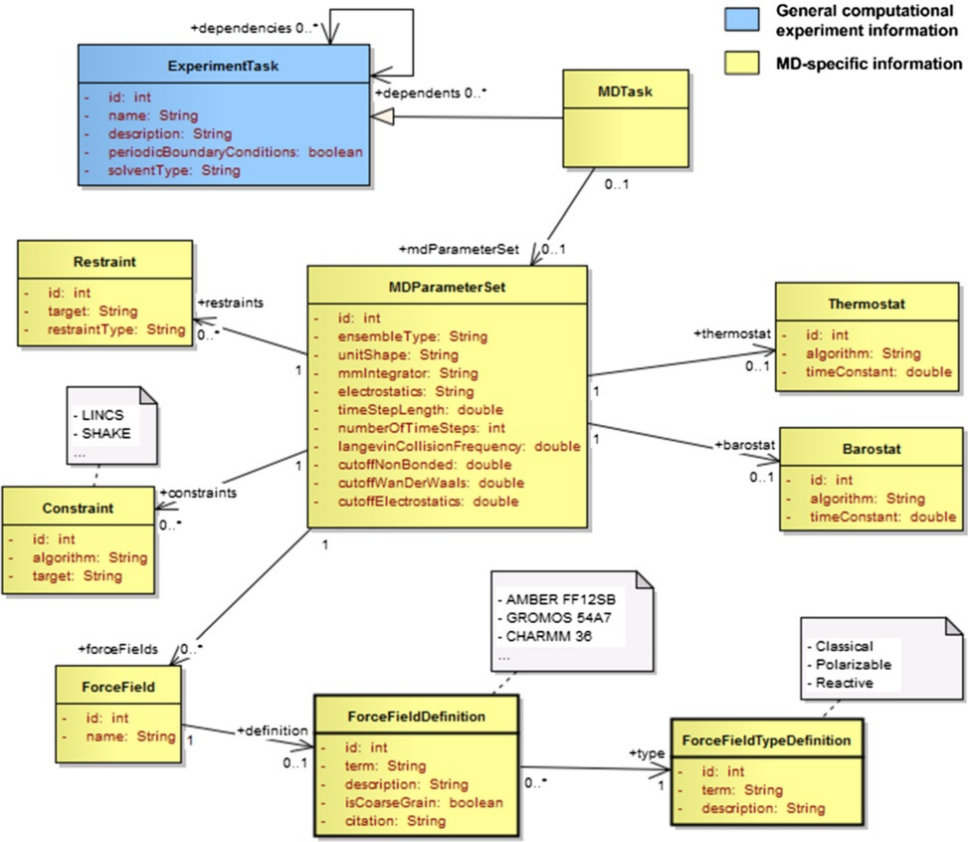
Description of MD tasks and parameter sets.

In molecular dynamics, the behaviour of the simulated system is governed by a *force field*: a parameterized mathematical function describing the potential energy of the system, and the parameters of the function, with dynamics propagated using Newton’s equations of motion and the atomic forces determined from the forces or first derivatives of the potential energy function. Different parameters will be used for different types of atoms (or group of atoms in the type of coarse grain dynamics). A given *force field* parameter set is usually adapted to particular types of residues in molecules (e.g. nucleobases in nucleic acids vs. amino acids in proteins). For a single *molecular dynamics task* multiple *force fields* and parameter sets can be used simultaneously. When simulating an explicit water-based solvent for example, the specific *force field* parameter set used to represent these water molecules (e.g. TIP3P, TIP4P, SPC/E [34]) will typically be different from the set used to parameterize the atoms of the solute or the ions. The *ForceField* class presented in Figure 8 represents instances of *force fields* referenced by a particular run while *ForceFieldDefinition* represents an entry from the dictionary listing known *force fields. Force field types* include classical, polarizable, and reactive force fields.

Molecular dynamics methods can be classified into more specific classes of methods. For example in stochastic dynamics (Brownian or Langevin Dynamics), extra parameters can be added to represent friction and noise [35]. In coarse-grain dynamics the force field is applied to groups of atoms rather than individual atoms. The differentiation between atomistic and coarse-grain dynamics is then achieved solely based on the type of force field used. In this model Langevin dynamics and coarse-grain dynamics are not represented by different types of tasks as they share the same *parameter set* as classic molecular dynamics. The *collision frequency* attribute used specifically by stochastic dynamics was added to the *MD parameter set* while a flag specifying whether the force field is atomistic or coarse grain is set in the force field dictionary.

Each *parameter set* can be associated to a *barostat* and a *thermostat* to define how pressure and temperature are constrained in the simulated system (Figure 8). The *ensemble type* (microcanonical, canonical, isothermal–isobaric, or generalized) can be defined directly in the *parameter set*. The model also includes the concepts of *constraints* and *restraints*. Both have a target (i.e. the list of atoms they apply to), which can be described by an atom mask or a textual description (e.g. ‘:WAT’, ‘water’). The type of *constraint* is defined by the algorithm used (e.g. SHAKE, LINCS) while the type of *restraint* is characterized by the property being restrained (e.g. bond, angle).

Enhanced sampling methods are gaining interest in the MD community as larger systems and longer time scales can be simulated faster than with classic approaches [36]. These methods usually involve the creation of multiple ensembles or replica that can be run in parallel (e.g. temperature replica-exchange, umbrella sampling). A dictionary of such methods was created to list popular enhanced sampling methods. At the core the runs based on these methods can still be represented with multiple *molecular dynamics tasks*. Depending on the method, the implementation, and the definition of the input files, the set of *MD tasks* corresponding to a given enhanced sampling run can be grouped into *processes* where each *process* represents either a separate ensemble/replica or a group of tasks run in parallel. For a replica exchange MD (REMD) run using 4 replicas, one could either group the 4 *MD tasks* into a single *process* representing the whole REMD run or 4 separate *processes* with a single *task* each.

**Figure 9:**
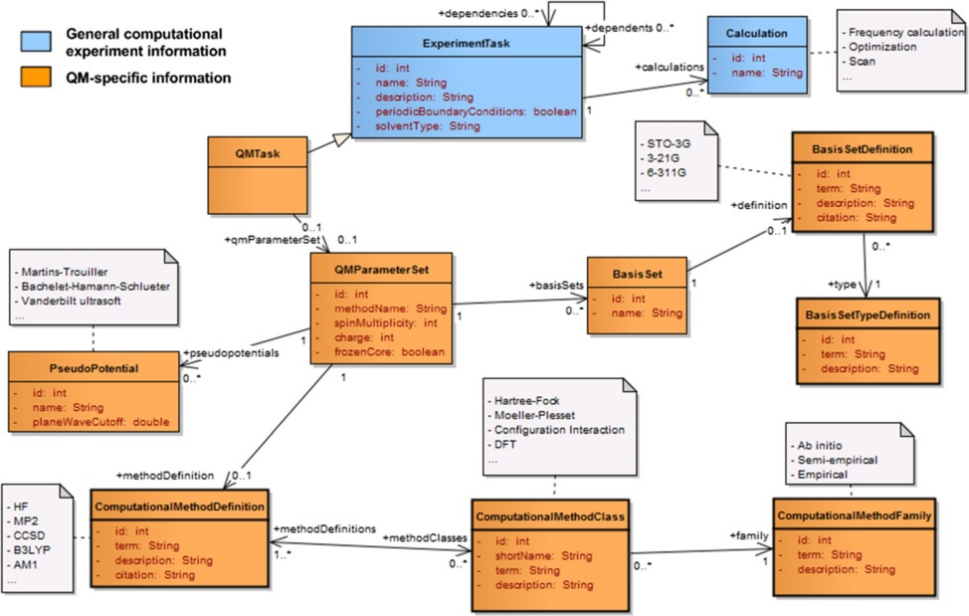
Description of QM tasks and parameters.

In quantum chemistry the two main elements that define the theory and approximations made for a particular run are the level of theory (or *QM method*) and the *basis set* (Figure 9). *Basis sets* provide sets of wave functions to create molecular orbitals and can be categorized into plane wave basis sets or atomic basis sets. They are defined in a dictionary (*BasisSetDefinition*). Different levels of theory are available to approximate the selected basis set and find a discrete set of solutions to the Schrödinger equation. Popular methods include Hartree-Fock and post-Hartree-Fock methods (e.g. Configuration Interaction, Møller-Plesset, Coupled-Cluster), multi-reference methods, Density Functional Theory (DFT), and Quantum Monte Carlo [37]. The classification of QM methods is not trivial because of the range of features dependent on the level of theory. For example, DFT method names typically correspond to the name of the exchange-correlation functional while semi-empirical method names provide a reference to the empirical approximations of the method. For this model we defined the concepts of *QM method*, *class* and *family*. At the highest level the *family* defines the method as “ab initio”, “semi-empirical”, or “empirical”. The *class* defines the level of theory for ab-initio methods (e.g. Hartree-Fock, Møller-Plesset, Configuration Interaction, DFT, Multi-reference), or the type of semi-empirical method (pi-electron restricted or all valence electron restricted). Note that one method can be part of multiple *classes* (e.g. Multi-reference configuration interaction, hybrid methods). At the lowest level the *method* name (e.g. MP2, B3LYP, AM1) corresponds to a specific method, as it would be called by a particular software package. Approximations of pure ab-initio quantum methods can be used to reduce the computational cost of the simulations. Typical approximations include the use of *frozen cores* to exclude inner shells from the correlation calculations and *pseudo-potentials* (effective core potentials) to remove the need to use basis functions for the core electrons. The use of such approximations is noted at the *QM parameter set* level.

Molecular dynamics methods can be “improved” by injecting quantum characteristics to the models (semi-classical methods). In ab-initio molecular dynamics, the forces for the system are calculated using full electronic structure calculations, avoiding the need to develop parameters a prior. In hybrid QM/MM, the simulation domain is divided into an MM space where the MD force field applies, and a QM space where molecular orbitals will be described. Different methods exist to treat the boundaries between the two spaces. The decomposition of runs into *tasks* and *parameter sets* make the integration of such methods possible and fairly straight forward. For example, one could create a new type of tasks for ab-initio molecular dynamics that would have at least two parameter sets: the *QM parameter set* defined earlier and a new parameter specific to ab-initio molecular dynamics that would define the time steps (number, length) and the type of method (e.g. Car-Parinello MD, Born-Oppenheimer MD).

#### Molecular system

**Figure 10:**
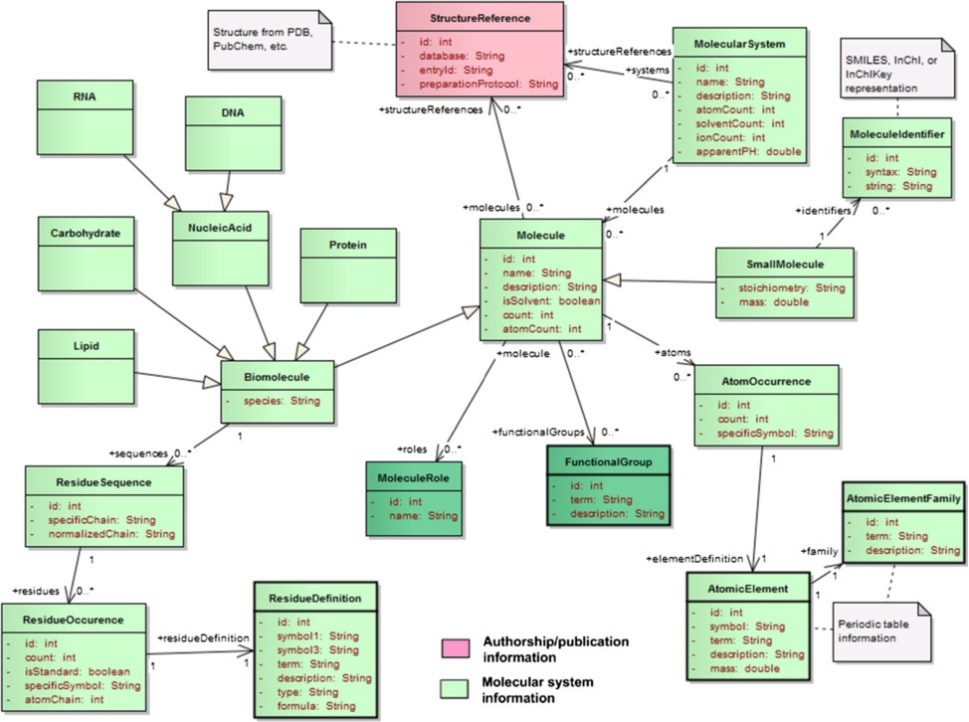
Decomposition of the molecular system into molecules with structural and biological features.

In this model a distinction is made between *biomolecules* (e.g. RNA, protein) and “*small molecules*” (Figure 10). Here we define a *small molecule* as a chemical or small organic compound that could potentially be used as a ligand. They are defined at the level of a single molecule while biomolecules are described by chains of residues. Typically, QM calculations will target *small molecules* while MD simulations will target larger *biomolecules* and ligand-receptor complexes. Properties such as molecular *weight* and *formula* are worth being tracked for small compounds but their importance is not that obvious when dealing with larger molecules.

Three dictionaries are necessary to provide definitions for *standard residues*, *atomic elements* (as defined in the periodic table), and *element families* (e.g. “Alkaline”, “Metals”). Note that here we minimize the amount of structural data by keeping track of occurrences of residues (*ResidueOccurrence)* and atom types (*AtomOccurrence*) in a particular molecule, rather than storing individual instances. For example, in the case of water, there will be a single entry for the hydrogen atom with a count set to 2, and another entry for the oxygen atom with a count set to 1. The same approach is used to keep track of the various molecules in the system. For example explicit solvent using water would be represented by the definition of the water molecule and the count of these molecules in the system. To enable searches of specific ligands a simple text representation of the compound is necessary. *Molecule identifiers* such as SMILES (Simplified Molecular-Input Line-Entry System [38]) or InChI (International Chemical Identifier [39]) strings can be associated to small molecules to enable direct molecule matching and similarity and substructure searches. The *residue sequence* is also available to search biomolecules based on an ordered list of residues. The *residue sequence* can be represented by two different strings: the original chain, or *specific chain*, as referenced in the input file defining the molecular topology, and a *normalized chain*. The *specific chain*, can potentially give more information about the individual residues within the context of the software that was used, and reference non-standard residues defined by the user. The *normalized chain* on the other hand uses a normalized nomenclature for the residue: one-letter codes representing either amino-acids or nucleobases. The *normalized chain* can be used to query the related molecule without prior knowledge about the software used, and enables advanced matching queries (e.g. BLAST [40]).

Both *residue* and *atom occurrences* can be given a *specific symbol*, which represents a software-specific name, usually referencing a computational model for the entity. In MD the *specific symbol* would be the force field atom type while in QM this would be used to specify which basis set should be applied.

The description of the biomolecules should include at least a generic type such as DNA, RNA or protein to classify the simulated molecules at a high level. Other biological information such as *species* (e.g. Mus musculus, Homo sapiens) and *molecule role* can be added as well. As defined by the Chemical Entities of Biological Interest (ChEBI [41]), each molecule can have one or multiple roles (application, chemical role, and/or biological role). This data element is very important as it would allow researchers to query molecules based on their function rather than their structure. On the other hand this type of information is not included in the raw simulation files, which means that it would have to be entered manually by the owner of the data. To avoid this one can imagine populating this information automatically by referencing external databanks that already store these attributes (e.g. Protein Data Bank [3]). This is reflected in this model by the *reference structure* concept, which keeps track of the database and the structure entry ID. If the topology of a simulated system is actually derived from a *reference structure* an extra field can be used to describe the protocol used to prepare the *reference structure* so that it serves as an input of the simulations. Possible steps include: choice of the specific model number if several are available in a single PDB entry or which PDB entry if multiple entries are possible, possible addition of missing residues from disordered regions, or specification of homology or other putative models.

#### Files and file system

**Figure 11:**
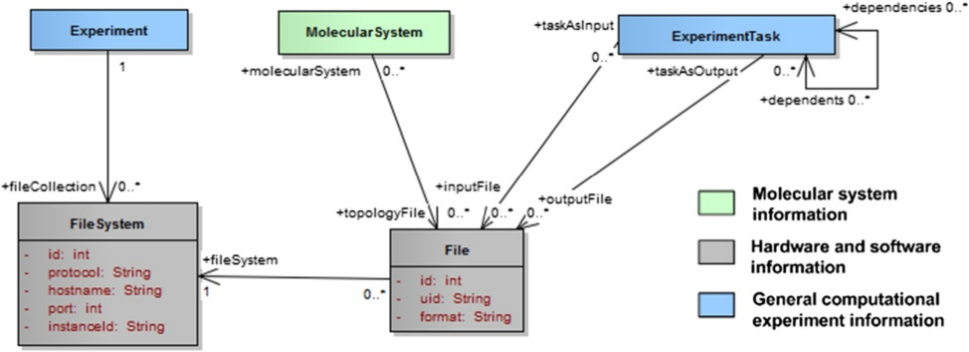
References to the file system and hosted files containing the raw data.

So far the description of the model focused on the data elements related to the experiment itself to explain why the different tasks were run and what they represent. Another important aspect of this model is the inclusion of a reference to the files (input and output) that contain the actual data being described. This is illustrated in Figure 11. Each *experiment* can be associated to one or several file collections stored on local or remote *file systems* (e.g. NFS, Amazon S3, iRODS server). For each of these collections no assumption should be made on the location or the implementation of the *file system*, therefore it is necessary to keep track of the type of file server and host information to find a route to the host and access the files using the right protocol and/or API. The individual files should be associated to the *tasks* they represent and a distinction between *input* (parameters and methods) and *output* (e.g. logs, trajectories) files should be made. The *topology files* should be associated to the *molecular system* instead. Note that in certain cases, especially for QM calculations, the topology and input parameters might be contained in the same file. Each file reference should at least contain a *unique identifier* (UID) within its host file system and a *format* specification.

#### Extended attributes

It is obvious that no single data model will be able to capture the needs of any lab running biomolecular simulations. The intent of this logical model is to provide a simple yet fairly exhaustive description of the concepts involved. To allow the addition of new properties, to provide more details about the experiment or to keep track of user- or lab-defined attributes, the notion of extended attribute can be introduced to the model. Each extended attribute would be an attribute-value-unit triplet referenced by a given class to extend its own attributes, as defined in the logical model. For example one user might want to keep track of the order of interpolation and the direct space tolerance for PME-based simulations. These parameters are currently not represented in the model, which only keeps track of the name of the electrostatics model (“PME”). To add these two parameters, one could add two extended attributes to the *MD parameter set* class (Figure 8) called “PME interpolation order” and “PME tolerance”.

From an object-oriented perspective, all the classes introduced in the logical model could inherit from a single superclass that would reference extended attributes, where each extended attribute would be an attribute-value-unit triplet with a possible link to a concept identifier defining the attribute in an existing terminology. From a database perspective, an extra table would be needed to store all the extended attributes. Such table would need the necessary columns to represent the attribute-value-unit triplet, a possible concept identifier, and the name of the table each attribute would extend. Although this is an easy way to gather all the extended attributes in a single table this approach is not rigorous from a relational approach. To allow SQL queries that do not involve injection of table names each table would have to be associated to an extra table storing its extended attributes.

### Summary

The logical model presented here defines a domain that should be sufficient to index biomolecular simulation data at the experiment level. In total over 60 classes were defined to represent the common data elements identified through the survey, along with new elements and dictionaries that should avoid data redundancy and facilitate queries using standard values. From a developer’s perspective this model provides some guidelines for the creation of a physical data model that would be more dependent on a particular technology, whether it is for the implementation of a database or an API. At a more abstract level the concepts introduced in this logical model provide a good starting point for the creation of a new terminology or ontology specific to biomolecular simulations.

### Dictionaries

#### Overview

The current list of dictionaries include: force field parameter set names and types (e.g. classical, polarizable), enhanced sampling methods, MD analysis functions, barostats, thermostats, ensemble types, constraint algorithms, electrostatics models, basis sets and their types, calculation types (e.g. optimization, frequency, NMR), residues, atomic elements (periodic table) and their families, functional groups, software packages, and chemical file formats. The list also includes a dictionary of computational methods (e.g. Langevin dynamics, MP2, B3LYP) with their class (e.g. MD, Perturbation Theory, DFT) and family (e.g. ab initio, semi-empirical, empirical). All these dictionaries are available for browsing and lookups at: http://ibiomes.chpc.utah.edu/dictionary/. Examples of dictionary entries are also provided in Additional file 6 (force fields) and Additional file 7 (computational methods).

#### Implementation

**Figure 12:**
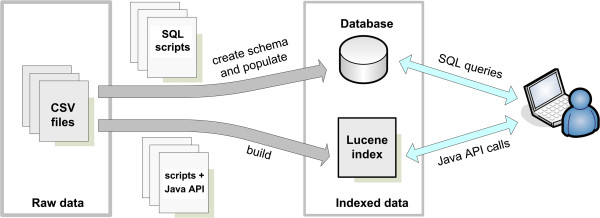
**Building process for the dictionaries.** Each dictionary can be either indexed via Apache Lucene for use via a Java API or loaded into a database to enable remote SQL queries.

All our dictionaries follow the same implementation method. The raw data is defined in CSV files and can be loaded into a database for remote queries and/or indexed using Apache Lucene [20] for local access via Java APIs (Figure 12). Apache Lucene is a text search engine written in Java that uses high-performance indexing to enable exact and partial string matching. Each CSV file contains a list of entries for a given dictionary with at least three columns representing: the identifiers, the terms (e.g. “QM/MM”), and the term descriptions (e.g. “Hybrid computational method mixing quantum chemistry and molecular mechanics”). More columns can be defined depending on the type of dictionary, either to represent extra attributes or to link to other dictionaries (foreign keys). For example the CSV file listing the QM method classes would have an extra column with the IDs of the associated QM method families. A set of SQL scripts was written to automatically create the database schema necessary to store the dictionaries and to load the CSV data into the tables. These scripts become very useful if one wants to integrate these dictionaries into a repository. Another script was written to automatically build the Lucene indexes. The script calls a Java API which parses the CSV files and uses the Lucene API to build the indexes. These indexes can then be used locally by external codes via the Lucene API, avoiding the need for static definitions of these dictionaries within the code or the creation of dependencies with remote resources such as a database. They should also help future developments of chemical file parsers and text processing tools for chemical information extraction from the literature (i.e. natural language processing). The Lucene-based dictionaries can be directly queried through a simple command-line interface. Additional file 8 demonstrates how one would look up a term using this program. This design is fairly simple and enables updates of the dictionary entries directly through the CSV files. One limitation is the lack of synonyms for the terms defined. To create richer lists it will be necessary to add an extra CSV file for each dictionary that would contain the list of all the synonyms and the ID of the associated terms. Successful implementations of terminologies in other domains, such as the UMLS (Unified Medical Language System [42]), should be used to guide the organization of the raw data and facilitate the integration of existing terminologies representing particular aspects of the biomolecular simulations (e.g. chemical data, biomolecules, citations).

#### Maintenance and community support

Until this point the development of the dictionaries has been restricted to an internal effort by our lab. To support the work of the community at large these dictionaries have to be extended and adjusted based on user feedback. For this purpose the dictionaries are now available on our project Wiki at http://ibiomes.chpc.utah.edu/mediawiki/index.php/Dictionary, which enables discussions and edits by identified users. This will serve as a single endpoint to draft new versions of the dictionaries. The source code for the dictionaries, including the CSV files, SQL scripts, and Java API, is available from *GitHub* at: https://github.com/jcvthibault/biosim-repository. Updates on the CSV files hosted there should occur according to the status of the dictionaries in the Wiki. With time we might find that a dedicated database with a custom user interface becomes necessary for a defined group of editors to update existing terms, add new entries, add new dictionaries, and keep track of changes (logs). In any case, the number of editors should be limited to a small group of experts, actively participating and working together [43,44].

## Discussion

In this paper we introduced a set of common data elements and a logical data model for biomolecular simulations. The model was built upon community needs, identified through a survey and refined internally. Elements described by the model cover the concepts of authorship, molecular system, computational method and platforms. Although the model presented here might not be complete, it integrates the methods that are the most significant for simulations of biomolecular systems: molecular dynamics, quantum chemistry and QM/MM. We introduced a new representation of the method landscape through method-specific parameter sets, which should allow the integration of more computational methods in the future. The addition of extended attributes to the model should enable customization by labs to fit their specific needs or represent properties that are currently not described by the model. The use cases presented here showed how the model can be used in real applications, to partially automate the creation of database schemas and generate XML descriptions. Multiple dictionaries, populated through reviews of online resources and literature, were implemented to supplement the model and provide developers with new tools to facilitate text extraction from chemical files and population of repositories. Although the current version of the dictionaries is fairly exhaustive they will become a powerful tool only if they are updated by the community. A missing piece in this model is a catalogue of available force field parameter sets and atom types that could be used to generate force field description files and serve as an input for popular MD software packages. The EMSL Basis Set Exchange [45] already offers something similar for basis sets, and provides a SOAP-based web service to access the data computationally.

While it is important to allow the whole community to provide input on the CDEs and dictionaries, eventually a consensus needs to be made by a group of experts representing the main stakeholders: simulation engine developers, data repository architects, and users. The creation of a consortium including users, developers and informaticians from the QM and the MD community could help formalize this process if such entity leads:

– Active polling, for example via annual surveys assessing the need for changes or additions in the CDEs, dictionaries, or the data model. Information about the respondents such as software usage, preferred computational methods (e.g. all-atom or coarse-grain MD, DFT) and target systems (e.g. chemical compounds, biomolecules) will provide more details for the development of more adequate recommendations for specialized communities.

– Monitoring of community discussions, which might take place on a dedicated online forum or a wiki such as the one introduced here

– Recurring creation and distribution of releases for the CDEs, dictionaries, and data model. The CDEs in particular should include at least 2 levels of importance (recommended or optional) to provide some criteria about the completeness of the data descriptors. A third level characterizing certain CDEs as mandatory might provide a standard for developers and data publishers to populate repositories.

Our current focus is on indexing data at the experiment level so that the associated collection of input and output files can be retrieved. While the CDEs can be used to tag individual files it is not clear yet how much metadata is necessary to enable automatic data extraction (e.g. extract properties for a single frame from a time series) and processing, and if such metadata can be extracted directly from the files without user input. The popularization of self-explanatory formats (e.g. NetCDF, CML) to store calculation results or MD trajectories would certainly help. The ongoing work within the ScalaLife programme should help the community move in this direction, while the data model presented here will provide a good framework to organize, describe, and index computational experiments comprising multiple tasks. By publishing this model and the list of CDEs we hope to encourage developments of new repositories for biomolecular simulations, whether they are part of an integrated computational environment (e.g. MDWeb) or not (e.g. iBIOMES). Both approaches should be addressed. On one hand, computational environments can easily keep track of the tasks performed during an experiment since the input parameters and topologies are directly specified within the environment. On the other hand, we still need to think about the developer community that works on new simulation engines, new force fields and new computational methods. They will still need to customize their simulation runs within more flexible environments where they can manually edit input files or compile new codes, and use local or allocated high-performance computing resources. Independent data repositories where data can be deposited through a publication process are probably more viable to overcome these requirements. Finally it is not clear who will be given access to these large computational environments or who will have the computational, storage, and human resources to deploy, sustain, and make such complex systems available to the community.

The goal of the proposed data model is to lay the foundations for a standard to represent biomolecular simulations, from the experiment level to the task level. For this purpose we wanted to integrate MD, QM, and QM/MM methods, all of which play a particular role in the field. Although classical MD is arguably the most popular approach for biomolecular simulations we believe that QM/MM approaches and ab initio MD for example will gain more and more interest as computational power increases and they should not be left out of a future standard. On the other hand we recognize that our model might not be as granular as others. The UMM XML [26] schema for example will be one of the first attempts to describe MD simulation input with enough granularity so that software-specific input files can be generated without information loss. Such effort is highly valuable for the MD community, and our data model will certainly evolve to integrate such models. Our short-term goal is to engage current repository and data model developers such as the ScalaLife (http://www.scalalife.eu/) and Mosaic (https://bitbucket.org/molsim/mosaic/wiki/Home) groups for MD and the Blue Obelisk (http://sourceforge.net/apps/mediawiki/blueobelisk/) group for QM and cheminformatics so that we can learn more about each other’s experience and try to align our effort towards an integrated data model that would fit the needs of the whole biomolecular simulation community.

## Conclusion

The framework presented here introduces a data model and a list of dictionaries built upon community feedback and selected experts’ experience. The list of core data elements, the models, and the dictionaries are available on our wiki at: http://ibiomes.chpc.utah.edu/mediawiki/.

As more implementation efforts are taken, the community will be able to assess the present data model more accurately and provide valuable feedback to make it evolve, and eventually support collaborative research. The list of desiderata for data model developments, for both conceptual and physical representations, should provide some guidance for the long task at play.

## Methods

This paper uses semi-structured interview methods to establish the community needs and preferences regarding biomolecular simulation data indexing and presentation. The common data elements were identified using an approach similar to [46], while the data model was built using standard modelling techniques to derive logical and physical models. Interested readers can find details of these techniques in [22].

## Abbreviations

MD: Molecular dynamics; MM: Molecular mechanics; QM: Quantum Mechanics; CDE: Common data elements; PME: Particle-Mesh Ewald.

## Competing interests

The authors declare that they have no competing interests.

## Authors’ contributions

JCT designed the data model and implemented the various examples and dictionaries. DRR worked on the description of trajectory and analysis data. TEC3 and JCF participated in the design of the data model and helped to draft the manuscript. All authors read and approved the final manuscript.

## Supplementary Material

Additional file 1 **Online survey extract.** This picture shows the section of the online survey assessing the computational platform-related data elements. Click here for file

Additional file 2 **Results of the survey.** This table presents results of the survey, based on the following Likert scale: 1 = “Not important at all”, 2 = “Not very important”, 3 = “Not sure, 4 = “Important”, 5 = “Very important”, N/A = “Not applicable”. N is the number of responses for a particular data element. The reported score is the average of points assigned by responders using the Likert scale. Click here for file

Additional file 3 **Summary of survey comments for each data element category.** This table summarizes the comments of the respondents for each category of data elements. The last column lists only the comments that were either proposing new data elements or changes to the original ones, and that were related to the data element category. The number of respondents N is the number of people who provided at least one comment for the associated category. Click here for file

Additional file 4 **Final set of common data elements.** This file contains several tables (one for each data element category) presenting the identified common data elements. Each data element can be described through multiple attributes. Recommended attributes are marked with an “R” and attributes that can be derived from other attributes are marked with a “D”. Attributes that should be associated to a unit are marked with a “U”. Click here for file

Additional file 5 **Analysis dataset description examples.** This document presents two examples of how the proposed data elements might be applied to common analysis data. Click here for file

Additional file 6 **Table representing the force field dictionary.** This table lists common parameter sets available for popular MD software packages. Each entry in the table is described through an ID (ID), a name (TERM), a description (DESCRIPTION), a possible list of citations (CITATION), a force field type ID (TYPE_ID), and whether the force field is coarse grain or not (IS_COARSE_GRAIN). Click here for file

Additional file 7 **Table representing the dictionary of computational methods.** This dictionary lists “specific” methods which can be referenced within an input file for a computational task. Each entry in the table is described through an ID (ID), a name (TERM), a description (DESCRIPTION), and a possible list of citations (CITATION). Click here for file

Additional file 8 **Lucene-based dictionary usage and lookup example.** This document demonstrates the use of the command-line interface to lookup terms in the Lucene-based dictionary. In this example the user searches terms that start with “AMBER FF”. The ‘-n 2’ option specifies that no more than 2 matches should be returned. Click here for file

## References

[B1] Šponer J Šponer JE Mládek A Banáš P Jurečka P Otyepka M How to understand quantum chemical computations on DNA and RNA systems? A practical guide for non-specialists Methods 2013 64 1 3 11 10.1016/j.ymeth.2013.05.025 23747334

[B2] Dror RO Dirks RM Grossman JP Xu H Shaw DE Biomolecular simulation: a computational microscope for molecular biology Annu Rev Biophys 2012 41 429 452 10.1146/annurev-biophys-042910-155245 22577825

[B3] Bernstein FC Koetzle TF Williams GJB Meyer EF Brice MD Rodgers JR Kennard O Shimanouchi T Tasumi M The protein data bank Eur J Biochem 2008 80 2 319 324 923582 10.1111/j.1432-1033.1977.tb11885.x

[B4] Simms AM Toofanny RD Kehl C Benson NC Daggett V Dynameomics: design of a computational lab workflow and scientific data repository for protein simulations Protein Eng Des Sel 2008 21 6 369 377 10.1093/protein/gzn012 18411223

[B5] Toofanny RD Simms AM Beck DA Daggett V Implementation of 3D spatial indexing and compression in a large-scale molecular dynamics simulation database for rapid atomic contact detection BMC Bioinformatics 2011 12 334 10.1186/1471-2105-12-334 21831299 PMC3166946

[B6] Meyer T D’Abramo M Hospital A Rueda M Ferrer-Costa C Perez A Carrillo O Camps J Fenollosa C Repchevsky D MoDEL (molecular dynamics extended library): a database of atomistic molecular dynamics trajectories Structure 2010 18 11 1399 1409 10.1016/j.str.2010.07.013 21070939

[B7] Ng MH Johnston S Wu B Murdock SE Tai K Fangohr H Cox SJ Essex JW Sansom MSP Jeffreys P BioSimGrid: grid-enabled biomolecular simulation data storage and analysis Future Gen Comput Syst 2006 22 6 657 664 10.1016/j.future.2005.10.005

[B8] Terstyanszky G Kiss T Kukla T Lichtenberger Z Winter S Greenwell P McEldowney S Heindl H Application repository and science gateway for running molecular docking and dynamics simulations Stud Health Technol Inform 2012 175 152 161 22942006

[B9] Adams S de Castro P Echenique P Estrada J Hanwell MD Murray-Rust P Sherwood P Thomas J Townsend J The quixote project: collaborative and open quantum chemistry data management in the internet age J Cheminform 2011 3 38 10.1186/1758-2946-3-38 21999363 PMC3206452

[B10] Phadungsukanan W Kraft M Townsend JA Murray-Rust P The semantics of Chemical Markup Language (CML) for computational chemistry: CompChem J Cheminform 2012 4 1 15 10.1186/1758-2946-4-15 22870956 PMC3434037

[B11] Murray-Rust P Rzepa HS Chemical markup, XML, and the World Wide Web. 4. CML schema J Chem Inf Comput Sci 2003 43 3 757 772 10.1021/ci0256541 12767134

[B12] Guha R Howard MT Hutchison GR Murray-Rust P Rzepa H Steinbeck C Wegner J Willighagen EL The Blue Obelisk-interoperability in chemical informatics J Chem Inf Comput Sci 2006 46 3 991 998 10.1021/ci050400b PMC4878861 16711717

[B13] de Jong WA Walker AM Hanwell MD From data to analysis: linking NWChem and Avogadro with the syntax and semantics of Chemical Markup Language J Cheminform 2013 5 1 25 10.1186/1758-2946-5-25 23705910 PMC3764975

[B14] Frisch MJ Trucks GW Schlegel HB Scuseria GE Robb MA Cheeseman JR Scalmani G Barone V Mennucci B Petersson GA Gaussian 09, Revision C.01 2009 Wallingford, CT: Gaussian, Inc

[B15] Valiev M Bylaska EJ Govind N Kowalski K Straatsma TP Van Dam HJJ Wang D Nieplocha J Apra E Windus TL NWChem: a comprehensive and scalable open-source solution for large scale molecular simulations Comput Phys Commun 2010 181 9 1477 1489 10.1016/j.cpc.2010.04.018

[B16] Thibault JC Facelli JC Cheatham TE III IBIOMES: managing and sharing biomolecular simulation data in a distributed environment J Chem Inf Model 2013 53 3 726 736 10.1021/ci300524j 23413948 PMC8489555

[B17] Rajasekar A Moore R Hou CY Lee CA Marciano R de Torcy A Wan M Schroeder W Chen SY Gilbert L iRODS Primer: integrated rule-oriented data system Synth Lect Inform Concepts Retrieval Serv 2010 2 1 1 143

[B18] Abouzied A Bajda-Pawlikowski K Huang J Abadi DJ Silberschatz A HadoopDB in action: building real world applications Proceedings of the 2010 ACM SIGMOD International Conference on Management of data 2010 Indianapolis, IN, USA: ACM 1111 1114

[B19] Thusoo A Sarma JS Jain N Shao Z Chakka P Zhang N Antony S Liu H Murthy R Hive-a petabyte scale data warehouse using hadoop Data Engineering (ICDE), 2010 IEEE 26th International Conference on 2010 Long Beach, CA, USA: IEEE 996 1005

[B20] Apache Lucenehttp://lucene.apache.org. Access January 2014

[B21] Herráez A Biomolecules in the computer: jmol to the rescue Biochem Mol Biol Educ 2006 34 4 255 261 10.1002/bmb.2006.494034042644 21638687

[B22] Tillmann G A practical guide to logical data modeling 1993 New York: McGraw-Hill

[B23] Foster I Kesselman C The Grid 2: Blueprint for a new Computing Infrastructure 2003 2 San Francisco, CA: Morgan Kaufmann

[B24] Saltz J Oster S Hastings S Langella S Kurc T Sanchez W Kher M Manisundaram A Shanbhag K Covitz P caGrid: design and implementation of the core architecture of the cancer biomedical informatics grid Bioinformatics 2006 22 15 1910 1916 10.1093/bioinformatics/btl272 16766552

[B25] Sun Y McKeever S Converting biomolecular modelling data based on an XML representation J Integr Bioinform 2008 5 2 doi:10.2390/biecoll-jib-2008-95. 10.2390/biecoll-jib-2008-95 20134068

[B26] Goni R Apostolov R Lundborg M Bernau C Jamitzky F Laure E Lindhal E Andrio P Becerra Y Orozco M ScalaLife white paper: standards for data handling ScalaLife, Scalable Software Services for Life Science 2013 Available at http://www.scalalife.eu/, access January 2014)

[B27] Case DA Cheatham TE 3rd Darden T Gohlke H Luo R Merz KM Jr Onufriev A Simmerling C Wang B Woods RJ The amber biomolecular simulation programs J Comput Chem 2005 26 16 1668 1688 10.1002/jcc.20290 16200636 PMC1989667

[B28] Hess B Kutzner C van der Spoel D Lindahl E GROMACS 4: algorithms for highly efficient, load-balanced, and scalable molecular simulation J Chem Theory Comput 2008 4 3 435 447 10.1021/ct700301q 26620784

[B29] Humphrey W Dalke A Schulten K VMD: visual molecular dynamics J Mol Graph 1996 14 1 33 38 10.1016/0263-7855(96)00018-5 8744570

[B30] Roe DR Cheatham TE III PTRAJ and CPPTRAJ: software for processing and analysis of molecular dynamics trajectory data J Chem Theory Comput 2013 9 7 3084 3095 10.1021/ct400341p 26583988

[B31] Michaud‒Agrawal N Denning EJ Woolf TB Beckstein O MDAnalysis: a toolkit for the analysis of molecular dynamics simulations J Comput Chem 2011 32 10 2319 2327 10.1002/jcc.21787 PMC3144279 21500218

[B32] Hospital A Andrio P Fenollosa C Cicin-Sain D Orozco M Lluis Gelpi J MDWeb and MDMoby: an integrated Web-based platform for molecular dynamics simulations Bioinformatics 2012 28 9 1278 1279 10.1093/bioinformatics/bts139 22437851

[B33] Svensson M Humbel S Froese RD Matsubara T Sieber S Morokuma K ONIOM: A multilayered integrated MO+ MM method for geometry optimizations and single point energy predictions. A test for Diels-Alder reactions and Pt (P (t-Bu) 3) 2+ H2 oxidative addition J Phys Chem 1996 100 50 19357 19363 10.1021/jp962071j

[B34] Jorgensen WL Tirado-Rives J Potential energy functions for atomic-level simulations of water and organic and biomolecular systems Proc Natl Acad Sci USA 2005 102 19 6665 6670 10.1073/pnas.0408037102 15870211 PMC1100738

[B35] Nadler W Brunger AT Schulten K Karplus M Molecular and stochastic dynamics of proteins Proc Natl Acad Sci USA 1987 84 22 7933 7937 10.1073/pnas.84.22.7933 3479772 PMC299450

[B36] Schlick T Molecular dynamics-based approaches for enhanced sampling of long-time, large-scale conformational changes in biomolecules F1000 Biol Rep 2009 1 51 20948633 10.3410/B1-51 PMC2948272

[B37] Cramer CJ Essentials of Computational Chemistry : Theories and Models 2004 2 Chichester, West Sussex, England ; Hoboken, NJ: Wiley

[B38] Weininger D SMILES, a chemical language and information system. 1. Introduction to methodology and encoding rules J Chem Inf Comput Sci 1988 28 1 31 36 10.1021/ci00057a005

[B39] McNaught A The IUPAC International Chemical Identifier: InChI – a new standard for molecular informatics Chem Int 2006 28 6 12 14

[B40] Altschul SF Gish W Miller W Myers EW Lipman DJ Basic local alignment search tool J Mol Biol 1990 215 3 403 410 2231712 10.1016/S0022-2836(05)80360-2

[B41] Degtyarenko K De Matos P Ennis M Hastings J Zbinden M McNaught A Alcántara R Darsow M Guedj M Ashburner M ChEBI: a database and ontology for chemical entities of biological interest Nucleic Acids Res 2008 36 suppl 1 D344 17932057 10.1093/nar/gkm791 PMC2238832

[B42] Bodenreider O The unified medical language system (UMLS): integrating biomedical terminology Nucleic Acids Res 2004 32 Database Issue D267 14681409 10.1093/nar/gkh061 PMC308795

[B43] Hardiker N Kim TY Bartz CC Coenen A Jansen K Collaborative development and maintenance of health terminologies AMIA Annu Symp Proc 2013 2013 Washington DC: American Medical Informatics Association 572 577 PMC3900200 24551359

[B44] Noy NF Tudorache T Collaborative ontology development on the (semantic) web AAAI Spring Symposium: Symbiotic Relationships between Semantic Web and Knowledge Engineering 2008 Stanford University, CA: AAAI Press 63 68

[B45] Schuchardt KL Didier BT Elsethagen T Sun L Gurumoorthi V Chase J Li J Windus TL Basis set exchange: a community database for computational sciences J Chem Inf Model 2007 47 3 1045 1052 10.1021/ci600510j 17428029

[B46] Kawamoto K Del Fiol G Strasberg HR Hulse N Curtis C Cimino JJ Rocha BH Maviglia S Fry E Scherpbier HJ Multi-national, multi-institutional analysis of clinical decision support data needs to inform development of the HL7 virtual medical record standard AMIA Annu Symp Proc 2010 2010 Washington DC: American Medical Informatics Association 377 381 PMC3041317 21347004

