# Supplementary figures and images for: Data model, dictionaries, and desiderata for biomolecular simulation data indexing and sharing
Source: J Cheminform. 2014 Jan 30;6:4. doi: 10.1186/1758-2946-6-4 (PMC3915074; doi:10.1186/1758-2946-6-4)

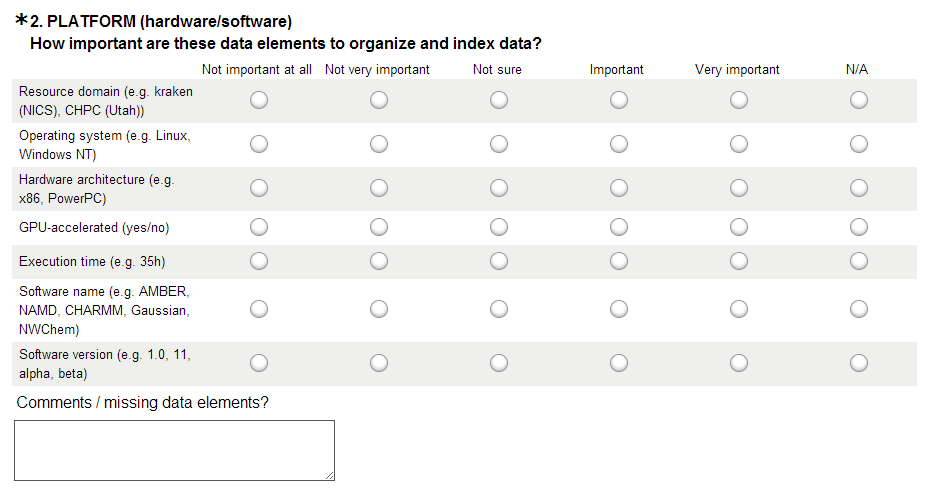

Supplement: Additional file 1 — Online survey extract. This picture shows the section of the online survey assessing the computational platform-related data elements. [file 1758-2946-6-4-S1.png]
