# Supplementary material for: Data model, dictionaries, and desiderata for biomolecular simulation data indexing and sharing
Source: J Cheminform. 2014 Jan 30;6:4. doi: 10.1186/1758-2946-6-4 (PMC3915074; doi:10.1186/1758-2946-6-4)
Supplement: Additional file 3 — Summary of survey comments for each data element category. This table summarizes the comments of the respondents for each category of data elements. The last column lists only the comments that were either proposing new data elements or changes to the original ones, and that were related to the data element category. The number of respondents N is the number of people who provided at least one comment for the associated category. [file 1758-2946-6-4-S3.docx]

**Summary of survey comments for each data element category**. The last column lists only the comments that were either proposing new data elements or changes to the original ones, and that were related to the data element category. The number of respondents N is the number of people who provided at least one comment for the associated category.

| **Data element category** | **N** | **Proposed data elements and changes** |
| --- | --- | --- |
| **Authorship** | 4 | - Missing: grant information  - Missing: timestamp / upload date |
| **Platform (hardware/software)** | 4 | - Missing: software compiled in single or double precision  - Change: GPU-accelerated is part of hardware architecture  - Missing: memory requirement, problems encountered during run |
| **Molecular system** | 5 | - Change: number of water molecules should be number of solvent molecules  - Missing: rigid parameters (e.g. some coordinate)  - Missing: water model is important |
| **Molecule** | 5 | - Missing: apparent pH  - Missing: information about the ligand (geometry and parameters)  - Missing: important functional groups |
| **Method (all)** | 7 | - Missing: broad classification of methods (empirical, semi-empirical, DFT, ab initio or combo of these) as well as static vs. dynamic.  - Change: convergence is both case dependent (energy vs. entropy vs. heat capacity...), and is also quite subjective.  - Change: convergence criteria would be difficult to track as the user will decide how to judge this  - Change: convergence is a moving target at best. Maybe there should be an overall convergence criteria metric, and if this minimum is met, it could be filed under "converged." |
| **MD methods** | 6 | - Missing: advanced sampling details, output details (e.g. steps per write), simulation scheme (whether this was a production run with such and such minimization and equilibration)  - Missing: restraints  - Missing: for PME, order of interpolation. For LINCS, order of expansion of the series.  - Missing: parallelization scheme |
| **QM methods** | 4 | - Missing: general property classifications (e.g. electron properties, pseudopotentials, frozen core)  - Missing: set of output properties available, and if QM method uses density functional theory related choices of exchange correlation and cut-offs |
