# Supplementary material for: Data model, dictionaries, and desiderata for biomolecular simulation data indexing and sharing
Source: J Cheminform. 2014 Jan 30;6:4. doi: 10.1186/1758-2946-6-4 (PMC3915074; doi:10.1186/1758-2946-6-4)
Supplement: Additional file 4 — Final set of common data elements. This file contains several tables (one for each data element category) presenting the identified common data elements. Each data element can be described through multiple attributes. Recommended attributes are marked with an “R” and attributes that can be derived from other attributes are marked with a “D”. Attributes that should be associated to a unit are marked with a “U”. [file 1758-2946-6-4-S4.docx]

**Final set of common data elements**

Each data element can be described through multiple attributes. Recommended attributes are marked with an “R” and attributes that can be derived from other attributes are marked with a “D”. Attributes that should be associated to a unit are marked with a “U”.

**Table 1. Data elements related to authorship**

| **Authorship (scope: experiment)** | **Attribute** | **U** | **R** | **D** |
| --- | --- | --- | --- | --- |
| Author | Full name (e.g. John Doe) |  | R |  |
|  | Institution name (e.g. university, company) |  |  |  |
|  | E-mail (e.g. john.doe@my.university.edu) |  |  |  |
| Citation | Identifier (e.g. DOI, PubMed ID) |  | R |  |
|  | URL |  |  |  |
| Publication based on the experiment results | Identifier (e.g. DOI, PubMed ID) |  | R |  |
|  | URL |  |  |  |
| Grant | Identifier |  | R |  |
|  | Source |  |  |  |
|  | Title |  |  |  |

**Table 2. Data elements related to the computational platform (hardware/software)**

| **Platform (scope: task)** | **Attribute** | **U** | **R** | **D** |
| --- | --- | --- | --- | --- |
| Computational environment | Resource domain (e.g. Kraken (NICS), Gordon (SDSC)) |  |  |  |
|  | Machine/supercomputer architecture (e.g. Cray XK7, IBM Blue Gene/Q) |  |  |  |
|  | Operating system (e.g. Linux, Windows NT) |  |  |  |
|  | CPU architecture (e.g. x86, PowerPC) |  |  |  |
|  | GPU architecture (e.g. Nvidia GTX 780) |  |  |  |
| Execution | Execution time (e.g. 35h) | U |  |  |
|  | Normal termination |  | R |  |
|  | Number of CPUs used |  |  |  |
|  | Number of GPUs used |  |  |  |
| Software | Name (e.g. AMBER, NAMD, CHARMM, Gaussian, NWChem) |  | R |  |
|  | Version (e.g. 1.0, 11, alpha, beta) |  | R |  |

**Table 3. Data elements related to the molecular system definition**

| **Molecular system** | **Attribute** | **U** | **R** | **D** |
| --- | --- | --- | --- | --- |
| System | Composition of the solvent (e.g. Water, Na+) |  | R |  |
|  | Number of solute molecules |  | R |  |
|  | Number of solvent molecules |  | R |  |
|  | Number of atoms in the system |  | R |  |
|  | Number of ions in the system |  | R |  |
|  | Apparent pH |  |  |  |
| Molecule | Type (e.g. Protein, RNA, DNA, chemical compound, nano-particle) |  | R |  |
|  | Name (e.g. Alanine, Sucrose, Tamoxifen) |  | R |  |
|  | Residue sequence (Amino-acid or nucleotide sequence) |  | R |  |
|  | Reference structure (e.g. PDB:1BIV, PubChem:2733526) |  | R |  |
|  | Molecular formula (e.g. C26H29NO) |  |  |  |
|  | Molecular weight (e.g. 371.51456 g/mol) | U |  |  |
|  | Whether it is part of the solvent or the solute |  | R |  |
|  | Main functional groups |  |  |  |

**Data elements related to the computational methods**

Table 4.a. Data elements common to any type of computational method

| **Method (scope: task)** | **Attribute** | **U** | **R** | **D** |
| --- | --- | --- | --- | --- |
| Method | General method name (e.g. MD, QM, Coarse-grain Dynamics, QM/MM) |  | R |  |
|  | Method reference citation (e.g. DOI, URL) |  |  |  |
|  | Whether the method simulates the dynamics of the system (Yes / No) |  |  |  |
| Boundary conditions | Type (Periodic, non-periodic) |  | R |  |
| Solvent model | Representation of the solvent (implicit, explicit, in vacuum) |  | R |  |
|  | Implicit solvent model name (e.g. GB HCT) |  |  |  |

Table 4.b. Data elements specific to molecular dynamics

| **MD (scope: task)** | **Attribute** | **U** | **R** | **D** |
| --- | --- | --- | --- | --- |
| Electrostatics model | Name (e.g. Cutoff, Classic ewald, PME, reaction field) |  | R |  |
| Unit shape | Type (e.g. cuboid, octahedron, cap, shell) |  |  |  |
| Ensemble | Type (e.g. NVE, NVT, NPT, Generalized) |  | R |  |
| Molecular mechanics integrator | Name (e.g. Euler, Runge-Kutta, Verlet, Leapfrog) |  |  |  |
| Constraint | Algorithm (e.g. LINCS, RATTLE, SHAKE, SETTLE) |  |  |  |
|  | Target |  |  |  |
| Restraint | Type (e.g. bond, angle) |  |  |  |
|  | Target |  |  |  |
| Force field | Name (e.g. AMBER FF 99, GROMOS 43A1 , ReaxFF) |  | R |  |
|  | Type (e.g. classical, polarizable, reactive) |  | R | D |
| Barostat | Name (e.g. Andersen, Berendsen, Parrinello-Rahman) |  |  |  |
|  | Time constant (e.g. 1000 fs) | U |  |  |
| Thermostat | Name (e.g. Berendsen, Nose, Nose-Poincare) |  |  |  |
|  | Time constant (e.g. 100 fs) | U |  |  |
| Time | Time step length (e.g. 1 picosecond) | U | R |  |
|  | Number of time steps |  | R |  |
|  | Total simulated time (e.g. 450 picoseconds) | U | R | D |
| Context of the run | Type (minimization, equilibration, or production) |  |  |  |
| Enhanced sampling method | Name (e.g. umbrella sampling, replica-exchange) |  |  |  |

Table 4.c. Data elements specific to quantum chemistry

| **QM (scope: task)** | **Attribute** | **U** | **R** | **D** |
| --- | --- | --- | --- | --- |
| QM method | Specific name (e.g. SCF, MP2, MP4, CCSD(T), B3LYP) |  | R |  |
|  | Family (e.g. Hartree-Fock, Moeller-Plesset, DFT, Configuration Interaction) |  | R | D |
| Basis set | Name (e.g. STO-3G, 6-31++G*, cc-pCDVZ) |  | R |  |
|  | Family (e.g. minimal, split-valence, plane-wave) |  |  | D |
| Spin multiplicity | Value |  |  |  |
| Total charge | Value |  |  |  |
| Froze core | Uses frozen core (yes/no) |  |  |  |
| Pseudo-potential | Implementation name (e.g. Martins-Trouiller) |  |  |  |
|  | Plane-wave cutoff | U |  |  |
| Convergence | Whether the run has converged (yes/no) |  |  |  |
|  | Convergence criteria (e.g. 10^-3) | U |  |  |
| Exchange-correlation functional | Name (e.g. B3LYP) |  |  |  |
