# Supplementary material for: Data model, dictionaries, and desiderata for biomolecular simulation data indexing and sharing
Source: J Cheminform. 2014 Jan 30;6:4. doi: 10.1186/1758-2946-6-4 (PMC3915074; doi:10.1186/1758-2946-6-4)
Supplement: Additional file 5 — Analysis dataset description examples. This document presents two examples of how the proposed data elements might be applied to common analysis data. [file 1758-2946-6-4-S5.docx]

## Analysis dataset description examples

Two examples of how the proposed data elements might be applied to common analysis data will be given. Note that currently the programs used in these examples do not necessarily report all of the metadata for these attributes; rather this is a recommendation of what metadata these programs could include in their output.

**Example 1**

The first example is the calculation of a distance between two atoms in a protein over the course of a molecular dynamics simulation totalling 101 ps in length, with the trajectory recorded at 1 frame per ps. The generated data set metadata can be as follows:

Analysis Name: Distance

Description: Distance in Cartesian space.

File: end-to-end.dat

Timestamp: Sat Nov 30 09:49:37 MST 2013

Filter on space: (Residue 2 atom CA), (Residue 12 atom CA)

Number Data Set Dimensions: 1

Dimension[1] size: 101

Number of variables: 2

Variable[1] units: picosecond

Variable[1] label: Time

Variable[1] type: float

Variable[1] uses dimension: 1

Variable[2] units: Angstrom

Variable[2] label: End to end distance

Variable[2] type: float

Variable[2] uses dimension: 1

Program: VMD

Version: V1.9.1

Command: distance “resid 2 and name CA” “resid 12 and name CA” 1 end-to-end.dat distr.dat

Note that there are actually two arrays sharing the same dimension, one (‘End to end distance’) containing the distance data and another (‘Time’) that holds the corresponding time steps of the data.

**Example 2**

The next example is the calculation of a mass-weighted coordinate covariance matrix for C-alpha atoms (12 atoms total) over 10 frames. Again there are two variables, but in this case the ‘Time’ variable would record which frames were used in generating the matrix, while the ‘matrix1’ variable is the 12x12 matrix itself.

Name: Mass-weighted Covariance Matrix

File: mwcovar.dat

Timestamp: Sat Nov 30 09:58:22 MST 2013

Filter on space: (All CA atoms)

Number Data Set Dimensions: 3

Dimension[1] size: 12

Dimension[2] size: 12

Dimension[3] size: 10

Number of variables: 2

Variable[1] units: picosecond

Variable[1] label: Time

Variable[1] type: float

Variable[1] uses dimension: 3

Variable[2] units: Angstrom*amu^0.5

Variable[2] label: matrix1

Variable[2] type: float

Variable[2] uses dimensions: 1, 2

Program: Cpptraj

Version: V13.12

Command: matrix mwcovar out mwcovar.dat name matrix1
