# Supplementary material for: Data model, dictionaries, and desiderata for biomolecular simulation data indexing and sharing
Source: J Cheminform. 2014 Jan 30;6:4. doi: 10.1186/1758-2946-6-4 (PMC3915074; doi:10.1186/1758-2946-6-4)
Supplement: Additional file 8 — Lucene-based dictionary usage and lookup example. This document demonstrates the use of the command-line interface to lookup terms in the Lucene-based dictionary. In this example the user searches terms that start with “AMBER FF”. The ‘-n 2’ option specifies that no more than 2 matches should be returned. [file 1758-2946-6-4-S8.docx]

**Command-line interface for Lucene-based dictionary lookups**

**Usage**

lucene-lookup.sh [options]

Options:

lookup -i <index-path> -t <term> [-f <lookup-field>] [-n <max-hits>]

list -i <index-path>

lookup: look up a term <term> in the Lucene index at <index-path> in a particular field <lookup-field>.

list: lists all the entries in the Lucene index at <index-path>

**Lookup example**

***Input command:***

lucene-lookup.sh lookup -i /tmp/dictionary_all -t "AMBER FF*" -n 2

***Console output:***

Lookup field: TERM

Term: AMBER FF*

Max hits: 2

Dictionary: /tmp/dictionary_all

Number of entries: 939

2 matches:

--------------------------------

[UID] 885

[ID] 1

[TERM] AMBER FF94

[DESCRIPTION] AMBER FF94 force field

[CITATION] Cornell et al. (1995), JACS 117, 5179-5197

[TYPE_ID] 1

[IS_COARSE_GRAIN] No

[ATTRIBUTE_TYPE] force_field

--------------------------------

[UID] 886

[ID] 2

[TERM] AMBER FF96

[DESCRIPTION] AMBER FF96 force field

[CITATION] Kollman (1996), Acc. Chem. Res. 29, 461-469

[TYPE_ID] 1

[IS_COARSE_GRAIN] No

[ATTRIBUTE_TYPE] force_field

--------------------------------
